# Tranexamic Acid for Reduction of Blood Loss in Patients with Extracapsular Proximal Femur Fractures: Systematic Review and Meta-Analysis of Randomized Clinical Trials

**DOI:** 10.3390/pharmaceutics18030374

**Published:** 2026-03-18

**Authors:** Irena Ilic, Ivan Stojadinovic, Branko Ristic, Milena Ilic

**Affiliations:** 1 Faculty of Medicine, University of Belgrade, 11000 Belgrade, Serbia; 2 Department of Surgery, Faculty of Medical Sciences, University of Kragujevac, 34000 Kragujevac, Serbia; 3 Department of Spinal Surgery, Clinic for Orthopedics and Traumatology, University Clinical Center Kragujevac, 34000 Kragujevac, Serbia; 4 Department of Traumatology, Clinic for Orthopedics and Traumatology, University Clinical Center Kragujevac, 34000 Kragujevac, Serbia; 5 Department of Epidemiology, Faculty of Medical Sciences, University of Kragujevac, 34000 Kragujevac, Serbia

**Keywords:** hip fractures, extracapsular fractures, intertrochanteric fractures, tranexamic acid, meta-analysis

## Abstract

**Background/Objectives**: Blood loss is a major concern in elderly patients undergoing hip fracture surgery. Tranexamic acid (TXA) is used to improve bleeding outcomes; however, randomized clinical trials (RCTs) report mixed findings, with some studies finding no improvements. This meta-analysis was conducted to evaluate the effectiveness of intravenous TXA in patients with extracapsular proximal femur fractures undergoing surgery. **Methods**: A systematic literature review was performed to identify relevant RCTs. Evaluated outcomes were total blood loss (TBL), hidden blood loss (HBL), change in hemoglobin (Hb), change in hematocrit (Hct), risk for transfusion and number of transfused units per patient. Review Manager 5.3 was used. **Results**: Twenty-five RCTs were included. TXA administration was associated with significant reductions in TBL (MD = −255.59 mL, 95% CI −306.50 to −204.68) and HBL (MD = −219.28 mL, 95% CI −286.93 to −151.62) compared with control. Patients receiving TXA had significantly smaller changes in Hb (MD = 0.65 g/dL, 95% CI 0.39–0.90) and Hct (MD = 4.22%, 95% CI 2.04–6.40). TXA significantly reduced the risk of transfusion (RR = 0.55, 95% CI 0.43–0.70) and number of transfused units per patient (SMD = −0.66, 95% CI −1.15 to −0.17). Subgroup analyses showed consistent effects. Sensitivity analyses confirmed robustness of results, except for the significance in reducing the number of transfused units when studies with ‘liberal’ transfusion thresholds were excluded. **Conclusions**: These findings show statistically significant improvements in bleeding outcomes with the use of intravenous TXA in patients with extracapsular proximal femur fractures undergoing surgery. Further high-quality RCTs are needed to standardize TXA timing and dosing.

## 1. Introduction

Over the last three decades, the number of people who have suffered a hip fracture has increased significantly by 92.7% [1]. Contrary to fractures by other anatomical locations, which have shown significant decreases in age-standardized burden estimates, the age-standardized incidence rate of hip fractures has remained stable over this period [1]. Hip fractures represent a significant public health issue due to the population aging and the associated burden both at the individual and society level [2]. Extracapsular hip fractures are localized distal to the joint capsule insertion, involve trochanteric and subtrochanteric fractures [3] and account for around 40% of all hip fractures [2].

Around 40% of patients with hip fractures have low hemoglobin levels on admission, with the progression of anemia being more pronounced in extracapsular fractures, and surgical procedures further leading to blood loss [4]. Additionally, subtrochanteric and intertrochanteric fractures of the femur are recognized as independent risk factors for the need for postoperative blood transfusions, which are in turn associated with poorer postoperative outcomes [4]. Extracapsular hip fractures are associated with a more extensive blood loss compared to intracapsular fractures, due to the soft tissue damage and resulting inflammatory response [5]. This is evident in hidden blood loss and more pronounced decrease in hemoglobin values [5].

Tranexamic acid (TXA) is a lysine analogue, which is used as an antifibrinolytic agent to reduce blood loss in orthopedic (total knee and hip arthroplasty, spine surgery), cardiac surgery, obstetrics and various other surgical and trauma settings [6]. While research has shown that the use of TXA in patients undergoing surgery for hip fracture significantly reduces total blood loss [7,8,9], some studies failed to confirm this [10,11]. Similarly, some studies did not find a significant reduction in the need for blood transfusions with the use of TXA [12,13,14,15,16] or in the number of transfused units [17,18].

Blood loss is of particular concern in this population because these individuals often already have some form of anemia, which can be worsened by the blood loss due to the fracture and surgery [19]. In addition to the possible early consequences of anemia that include delirium, impaired muscle strength and increased disability later in the recovery period, these persons have a higher risk of falls, re-admission and death [20,21,22,23]. At the same time, many of the patients undergoing hip fracture surgery require blood transfusion. While necessary, it might also contribute to the increased risk of infections both local and systemic, which can increase the risk of mortality in this often-frail population [24]. In addition to the worse outcomes, all of these complications can also increase the length of stay and finally the costs [25].

So far, meta-analyses have often inconsistently assessed this problem, sometimes without detailed subgroup analyses by hip fracture type or type of surgery [26,27], or without stratification by route of TXA administration [28,29]. Previous meta-analyses, which looked specifically into extracapsular fractures, have shown that TXA improves bleeding outcomes [28,30,31]; however, the evidence is not consistent [29,32,33], warranting a comprehensive and up-to-date analysis.

Given that recent projections indicate that the number of hip fractures per year will double by 2050 [34] and given the inconsistent findings from randomized clinical trials and previous meta-analyses, an updated evidence synthesis on the use of TXA for blood loss management in this population undergoing surgical management is warranted. The aim of this study was to determine the effects of the intravenous use of tranexamic acid on blood loss and the risk of transfusion in patients with extracapsular proximal femur fractures.

## 2. Materials and Methods

### 2.1. Study Design

A systematic review with meta-analysis was conducted in accordance with the Preferred Reporting Items for Systematic Reviews and Meta-Analysis (PRISMA) 2020 guidelines [35] (Table S1). The study protocol was not pre-registered.

### 2.2. Literature Search Strategy

Literature searches were conducted in PubMed, Web of Science (WOS), Scopus and Cochrane Central Register of Controlled Trials (CENTRAL), looking for articles from inception until 22 August 2025. There were no language restrictions, and no database filters were applied. The search strings used for each database are listed in Supplementary Table S2. Additionally, reference lists of relevant articles, including identified primary studies and reviews, were hand-searched. Two authors independently searched the databases, screened the titles and abstracts and checked the full-texts of papers potentially meeting the eligibility criteria and assessed them in line with the specified inclusion and exclusion criteria. In case of any disagreements, a third author helped reach a consensus.

### 2.3. Eligibility Criteria

Articles were considered for inclusion if they were designed as randomized clinical trials (RCT) conducted in the population of patients undergoing surgical treatment (intramedullary nailing or dynamic hip screw) for the management of extracapsular proximal femur fractures (intertrochanteric, peritrochanteric, subtrochanteric), if they provided comparison estimates between the intervention group receiving TXA intravenously and the control group receiving placebo or saline (no TXA). Articles were excluded if they were non-randomized trials, had no control group, did not involve intravenous administration of TXA, considered intracapsular fractures and arthroplasty, were not conducted in humans, were case reports, meta-analyses, reviews, letters, editorials, or conference abstracts. Pathological fractures (bone metastases, myeloma, osteoporosis other than age-related) were not considered. In the case of multiple articles reporting the results of the same RCT, the most recent publication or the publication with the most available data was included.

### 2.4. Outcomes of Interest

The efficacy outcomes that were considered in this meta-analysis were total blood loss (TBL), hidden blood loss (HBL), change in hemoglobin (Hb) levels, change in hematocrit (Hct) levels, need for transfusion and the number of transfused units per patient.

When outcomes for the changes in hemoglobin and hematocrit levels were reported at multiple time points across the considered primary studies, a single time point per study (identical for both groups) was selected to ensure comparability between intervention and control groups and minimize bias introduced by heterogeneous timing of measurements across studies. Specifically, in these cases, data was extracted from the time point with the longest follow-up as a clinically important time point [36]. Namely, early postoperative values may be substantially influenced by acute hemodilution, perioperative fluid shifts and the fluid retention after surgery which could lead to possibly underestimating or misrepresenting the actual changes [19], while later measurements better capture all processes which contribute to hemoglobin and hematocrit levels and provide a more meaningful estimate of the true treatment effects and correlate better with functional recovery and outcomes [37].

In trials with multiple TXA intervention arms, a single TXA arm was selected to avoid unit-of-analysis errors. Specifically, for the main analysis, the arm with a single dose of TXA was selected. In sensitivity analyses, the same analyses were conducted by selecting the trial’s arm with multiple TXA doses.

### 2.5. Risk of Bias Assessment

The risk of bias for each included study was estimated using the Cochrane’s Risk of Bias 2 (RoB 2) tool [38]. The RoB 2 tool addresses five domains, i.e., causes of bias arising from randomization, deviations from intended allocation, missing outcome data, measurement of the outcome and selection of the result that is reported. The judgments for the risk-of-bias can be low risk, some concerns and high risk of bias. Plots for visualization of the risk of bias assessments were created using the robvis tool [39].

While the RoB 2 tool is intended to be used for each considered result, we have combined the risk of bias assessment for outcomes for which the method of assessment and reporting are nearly identical, making it plausible to provide one assessment—i.e., for TBL and HBL—and for change in Hb and change in Hct. For other outcomes, separate assessments were made.

### 2.6. Statistical Analysis

Data on outcomes of interest (means, standard deviations (SD), number of events, rates) were extracted from primary studies. For continuous outcomes, the mean difference (MD) with 95% confidence interval (95% CI) was computed as the pooled estimate, while for dichotomous outcomes, the risk ratio (RR) with 95% CI was calculated. A random effects model was applied [40].

For the missing input values for certain outcomes of interest (i.e., change in Hb, change in Hct), such as SD of change, SDs were imputed assuming a correlation coefficient r of 0.5 [36]. The robustness of results was assessed by conducting sensitivity analyses for all outcomes under the assumptions of r = 0.3 and r = 0.7. Computations were performed using Meta-Analysis Accelerator [41]. In case of a study only reporting an outcome of interest (TBL) as mean and range, the conversion method proposed by Hozo et al. [42] was used to estimate SDs.

Presence of heterogeneity was checked using the chi-square test, with *p* < 0.1 indicating the presence of significant heterogeneity. The I^2^ statistic was used to quantify heterogeneity, with I^2^ values of 0–40% indicating possibly not important heterogeneity, 30–60% possibly moderate, 50–90% possibly substantial and 75–100% considerable [36]. Subgroup analyses were conducted by geography (studies conducted in China vs. outside China) and by transfusion threshold (‘restrictive’, i.e., Hb < 7–8 g/dL vs. ‘liberal’, i.e., Hb < 9–10 g/dL), for all outcomes where the number of studies allowed. Sensitivity analyses were performed to assess the robustness of pooled estimates. These included the leave-one-out method, exclusion of studies with high risk of bias, exclusion of outlier studies identified on visual inspection of funnel plots, exclusion of studies with mean age of participants < 65 years, exclusion of studies with large weights, and exclusion of studies that did not clearly define transfusion thresholds or used high thresholds (Hb ≥ 9 g/dL) [43]. Additionally, analyses were repeated using alternative r correlation coefficients (0.3 and 0.7) for imputation of SDs of mean change for Hb and Hct (Figures S1 and S2) and using data from the intervention arm with multiple TXA doses from multi-arm trials (Figures S3–S5).

Visual inspection of funnel plots was used to check for publication bias. Graphical presentation of pooled estimates was conducted via forest plots.

All analyses were performed in the Review Manager software (RevMan version 5.4.1, Copenhagen, Denmark, The Nordic Cochrane Center, The Cochrane Collaboration, 2020) [44]. A *p* value < 0.05 was considered statistically significant.

## 3. Results

### 3.1. Search Results

**Figure 1:**
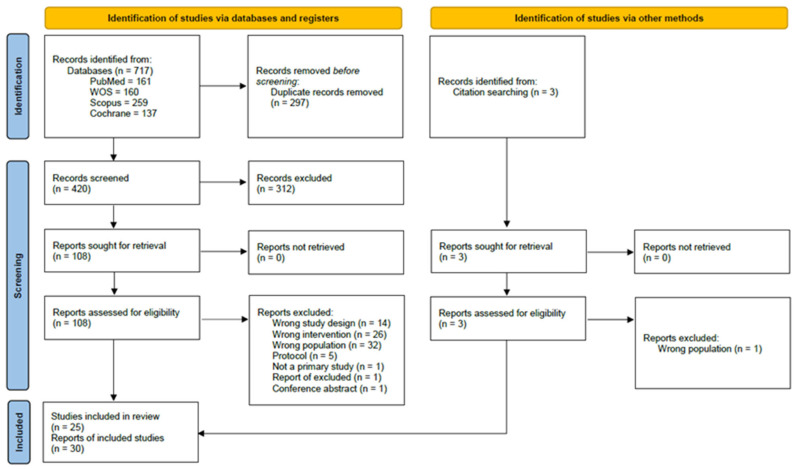
Flow diagram of literature search.

The literature search of databases and through citation searching yielded 720 results in total. After removing duplicates and screening titles and abstracts, there were 111 reports that were sought in full-text and assessed against predefined inclusion and exclusion criteria. Finally, 25 studies [7,8,9,10,11,12,13,14,15,16,17,18,45,46,47,48,49,50,51,52,53,54,55,56,57] were included in the quantitative synthesis of results. Detailed search results are shown in Figure 1.

### 3.2. Study Characteristics

Out of the 25 included studies, more than half were conducted in China (*n* = 13), followed by Pakistan (*n* = 3), the United States, Iran and India (each *n* = 2), and Turkey, Greece and Denmark (each *n* = 1). The sample size of studies ranged from 30 to 200, totaling 2357 patients that were considered in the meta-analysis across all included studies. Fracture types were predominantly intertrochanteric or peritrochanteric femoral fractures (AO/OTA 31-A1-A3). Surgical fixation techniques included intramedullary/cephalomedullary nails (proximal femoral nail, proximal femoral nail antirotation, trochanteric fixation nail advanced) and extramedullary fixation with dynamic hip screws or plates. Most studies reported a higher proportion of female patients. Mean age in both intervention and control groups generally ranged from about 65 to 85 years, with the majority of study populations comprising elderly patients. Detailed study characteristics are shown in Table 1.

### 3.3. Risk of Bias of Included Studies

Most of the included studies were judged to have low risk of bias; several studies demonstrated some concerns, mostly in the domain related to bias arising from the randomization process, and only two studies showed overall high risk of bias (Figures S6–S9).

### 3.4. Total Blood Loss and Hidden Blood Loss

**Figure 2:**
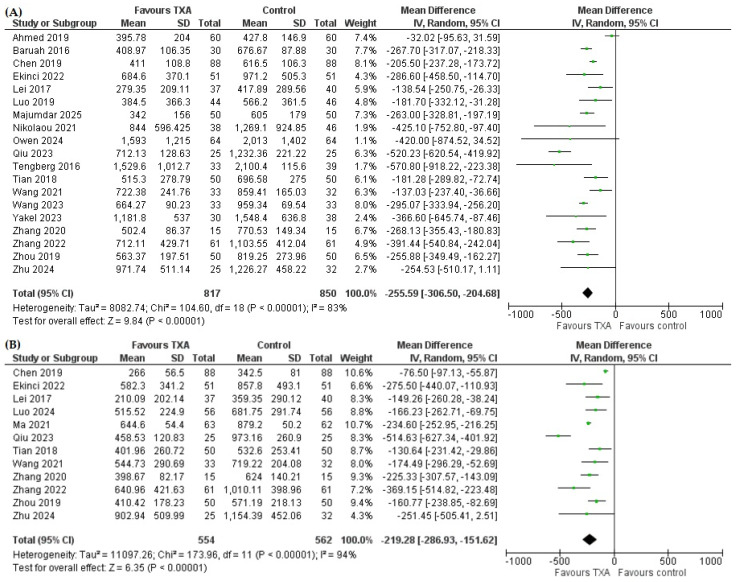
Forest plot for the effect of tranexamic acid (TXA) versus control on (**A**) total blood loss [7,8,9,10,11,14,15,16,17,18,46,47,48,49,50,51,54,56,57] and (**B**) hidden blood loss [7,8,13,16,18,47,48,49,50,52,54,57].

Nineteen studies reported TBL [7,8,9,10,11,14,15,16,17,18,46,47,48,49,50,51,54,56,57], comprising 817 patients in the TXA and 850 in the control group. The pooled analysis demonstrated that TXA significantly reduced TBL compared with control. The mean difference was −255.59 mL (95% CI −306.50 to −204.68, *p* < 0.00001) (Figure 2A).

There were twelve studies that reported HBL [7,8,13,16,18,47,48,49,50,52,54,57] with 554 patients who received TXA and 562 in the control group. As shown in Figure 2B, the TXA group experienced a significantly lower HBL compared to the control group (MD= −219.28 mL, 95% CI −286.93 to −151.62, *p* < 0.00001).

### 3.5. Hb and Hct Change

**Figure 3:**
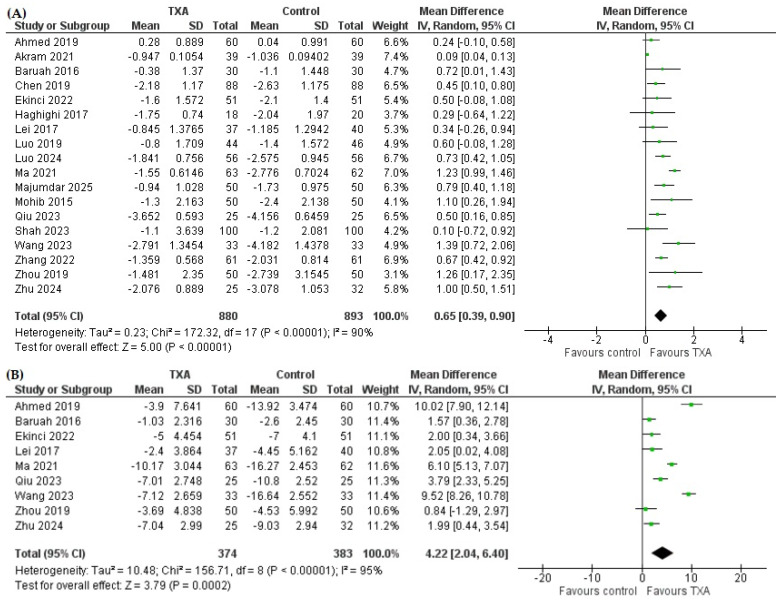
Forest plot for the effect of tranexamic acid (TXA) versus control on change in (**A**) hemoglobin [7,10,12,13,14,15,16,18,45,46,47,49,50,51,52,53,55] and (**B**) hematocrit [7,10,15,16,18,46,49,50,52].

Patients who received TXA had a significantly lower decline in Hb compared to the control group (Figure 3A). The mean difference of change in Hb was 0.65 g/dL (95% CI 0.39 to 0.90, *p* < 0.00001) in favor of the TXA group, indicating a smaller Hb decrease compared with controls.

The mean difference in Hct change was 4.22% in favor of TXA (95% CI 2.04 to 6.40, *p* = 0.0002), indicating a smaller decrease in Hct compared with controls (Figure 3B).

### 3.6. Transfusion Rate and Transfused Blood Units

**Figure 4:**
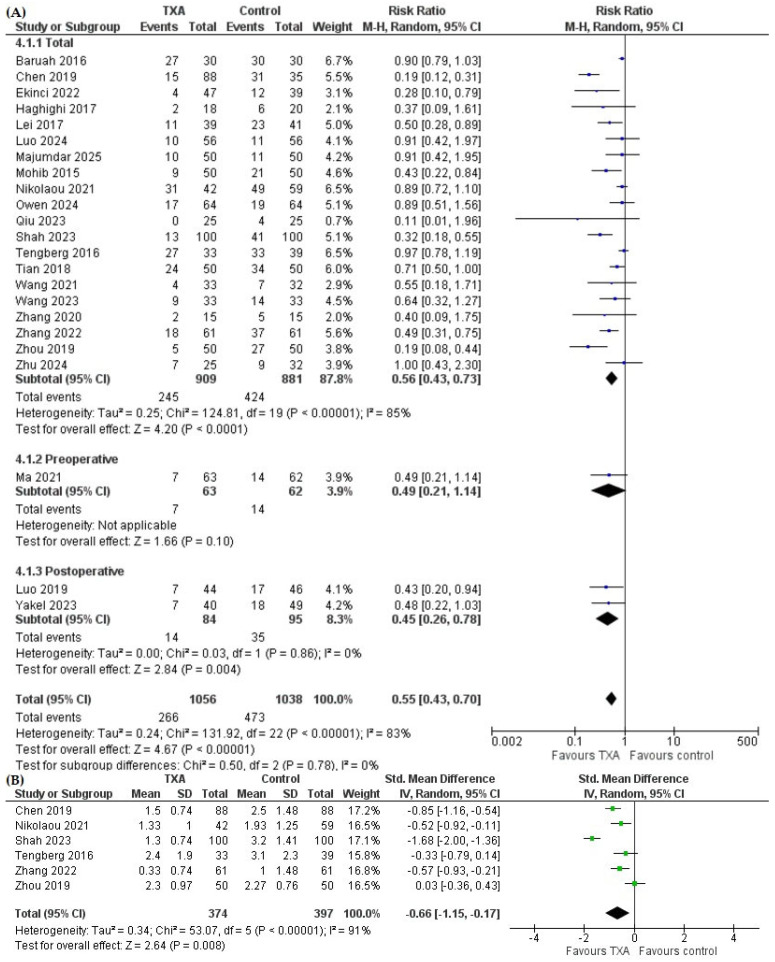
Forest plot for the effect of tranexamic acid (TXA) versus control on (**A**) transfusion rate [7,8,9,11,12,13,14,15,16,17,18,46,47,48,49,50,51,52,53,54,55,56,57] and (**B**) number of transfused units per patient [8,17,18,47,55,57].

Tranexamic acid significantly reduced the risk of blood transfusion compared with control (RR = 0.55, 95% CI 0.43 to 0.70, *p* < 0.00001). In subgroup analyses, TXA significantly reduced overall transfusion rate (RR = 0.56, 95% CI 0.43 to 0.73, *p* < 0.0001) and postoperative transfusion rate (RR = 0.45, 95% CI 0.26 to 0.78, *p* = 0.004), while one study that reported preoperative transfusion rate did not find a significant difference; subgroup analyses according to transfusion reporting did not reveal significant subgroup differences (*p* = 0.78) (Figure 4A).

Pooled analysis of six studies reporting the number of transfused units per patient (Figure 4B) showed that TXA was associated with a significantly lower number of transfused units per patient compared with controls (SMD = −0.66, 95% CI −1.15 to −0.17, *p* = 0.008).

### 3.7. Subgroup and Sensitivity Analyses

Subgroup analyses showed that the effect of TXA was consistent between studies conducted in China and outside China for all observed outcomes (Table 2). Additionally, the difference in Hct change remained statistically significant only in studies conducted in China. Subgroup analysis by transfusion threshold revealed a significant subgroup difference (*p* = 0.04) with a larger treatment effect of TXA on reducing the risk for transfusion when the ‘restrictive’ threshold was applied compared with ‘liberal’.

The magnitude of the effect of adding TXA varied slightly across sensitivity analyses, and the overall direction of the effect remained consistent, indicating that the results were robust to the applied assumptions and exclusions. Statistical significance was preserved across all outcomes and sensitivity analyses except for one: sensitivity analysis of the number of transfused units, where the pooled effect after excluding studies with ‘liberal’ transfusion thresholds was SMD = −0.47 (95% CI −0.98 to 0.03, *p* = 0.07).

### 3.8. Publication Bias

Visual inspection of funnel plots (Figure S10) did not suggest clear evidence of publication bias.

## 4. Discussion

This meta-analysis demonstrates that TXA significantly and meaningfully reduces total and hidden blood loss, transfusion rate and the number of transfused units in patients undergoing surgery for extracapsular proximal femur fractures. In addition, TXA significantly attenuated declines in hemoglobin and hematocrit. The findings were consistent across most subgroup and sensitivity analyses, supporting the robustness of observed treatment effects.

Administration of TXA resulted in a reduction of TBL, which was not only statistically significant, but the observed difference of approximately 256 mL compared to controls can also be considered clinically significant, especially in the elderly, frail population with limited physiological reserve [58]. Among individual studies that reported this outcome, some trials showed a lack of statistically significant benefit in the reduction of TBL with the use of TXA. Discrepancies between statistical significance reported in individual trials and results obtained using standardized meta-analytical calculations based on reported data may occur, and such differences may reflect variations in statistical methodology, limited statistical precision or reporting practices in individual studies [10], as was indicated in the RoB 2 assessments of this meta-analysis. Further on, Owen et al. [11] reported a lower cumulative estimated blood loss in patients who received TXA compared to controls, with a mean difference of 420 mL, but this difference was not statistically significant. The authors of the study have noted that their trial was designed to detect a meaningful difference in transfusion rates, but their post-hoc power analysis showed that the trial was significantly underpowered. Additionally, Owen et al. [11] reported that they did not exclude patients based on preoperative use of antiplatelet or anticoagulant drugs. Also, the trial reported deviations from the administration protocol: perioperatively, both groups, including the control group, received TXA, and all patients were included in the analysis after a comparative distribution of the primary outcomes analysis. Consequently, the risk of bias assessment performed in this meta-analysis reflected this by judging the trial as having a high risk of bias, and further exploring this in sensitivity analyses. Hidden blood loss, often underrecognized in hip fracture surgery, was also significantly reduced, suggesting that TXA might mitigate microvascular bleeding and fibrinolysis not captured by intraoperative measurements alone. This finding suggests that the benefit from TXA goes beyond visible surgical bleeding. Consistent with reductions in blood loss, patients receiving TXA experienced significantly smaller declines in Hb and HCT levels compared to controls. Research shows that a reduction in hemoglobin is substantial in patients with extracapsular hip fractures (mean 20.2 g/L, range 0–49 g/L) [59]; thus, a mean difference in Hb reduction of 0.65 g/dL associated with the administration of TXA found in this study can be considered relevant in this population. This is particularly important given the elderly population, where even modest drops in Hb may increase the risk of transfusion, delay mobilization and affect postoperative outcomes [60]. Patients with extracapsular hip fractures experience traumatic bleeding into the soft tissue following the fracture, in addition to visible and overall blood loss due to surgical procedures, and these should be considered in the context with the changes in Hb and Hct levels, and often already existing anemia in the elderly, which combined support the clinical relevance of the shown effects of TXA on reducing TBL and HBL and reducing the drop in Hb and Hct, alongside associated transfusion risk in elderly patients.

Tranexamic acid significantly reduced the risk of blood transfusion by 45% and the number of transfused units per patient compared with the control. This finding is particularly relevant given the known association between transfusion and adverse outcomes in elderly orthopedic patients, i.e., infection, volume overload and prolonged hospitalization; thus, administration of TXA could have the potential to reduce transfusion-related risks [22,23,24]. Subgroup analyses by transfusion thresholds and geographic location were performed because these factors are known to influence transfusion practices, clinical decision making, clinical outcomes and observed treatment effects in primary studies and have been identified as important sources of heterogeneity in previous meta-analyses [31,61]. Transfusion thresholds represent a key clinical decision parameter that can affect outcome, while geographic differences may reflect variability in clinical protocols, timing and dose of TXA administration, perioperative management, patient characteristics and healthcare system factors. The magnitude of benefit varied depending on transfusion thresholds, with a more pronounced effect observed in studies which applied restrictive transfusion criteria, which was consistent with previous research which showed a significant reduction in transfusion rate: RR = 0.486 (95% CI 0.377–0.626) across studies with low transfusion threshold (7–8 g/dL) and RR = 0.694 (95% CI 0.493–0.978) among studies with a high threshold (>8 g/dL) [26]. The Transfusion Trigger Trial for Functional Outcomes in Cardiovascular Patients Undergoing Surgical Hip Fracture Repair (FOCUS) investigated whether higher thresholds for blood transfusion improve outcomes compared with restrictive thresholds and found no evidence of differences in functional outcomes, morbidity or mortality [62].

Subgroup analyses revealed an absence of geographic variability in the direction of effect, with some variations in its magnitude, whereas stronger effects were observed in studies conducted in China. Possible explanations include differences in characteristics of the study population, treatment protocols or some methodological differences, including different (i.e., earlier) timing of TXA administration and sequential use. Persistence of significant differences in hematocrit reduction with the use of TXA, primarily in studies conducted in China, might suggest differences or changes in care. Namely, research shows that treatment protocols that include preoperative nutrition management when combined with TXA improve outcomes [63], and that undernutrition can hinder the effects of TXA on blood loss and rates of blood transfusion [64]. Additionally, China has seen improvements in the implementation of the co-management care model for older patients with hip fracture [65], which was previously identified as significantly limited [66]. Although the locations of studies conducted outside of China were heterogeneous, small numbers did not allow for different meaningful groupings by geography. Still, the results suggest that assuming generalizability of findings for change in Hct across healthcare systems and locations should be done with caution. One meta-analysis of the use of TXA in hip fractures also found a significant difference in blood transfusion rates between China and non-China studies (RR = 0.45 and 0.68, respectively) [61]. Further, two meta-analyses demonstrated that while there were no subgroup differences between China and non-China studies, the effects on TBL were more pronounced in studies conducted outside China [31,61]. Possible reasons for this difference compared to the present meta-analysis could involve inclusion of both intra- and extra-capsular fractures, considering both IV and topical administration route, as well as a smaller number of analyzed studies.

Sensitivity analyses largely confirmed the robustness of results, with a consistent direction of effect across outcomes. It was notable that once studies with ‘liberal’ thresholds for transfusion were excluded, the reduction in the number of transfused units was no longer statistically significant. This suggests that the impact of TXA on the need for transfusion units could depend on clinical decision-making and existing operational practices, but it should also be noted that this result might reflect the small number of studies in the analysis rather than a true absence of effect. Previous research has shown that blood transfusion thresholds can at least in part explain the heterogeneity among considered outcomes [61]. The updated guidelines of the Association for the Advancement of Blood and Biotherapies (AABB) recommend a restrictive blood transfusion strategy, specifically 7–8 g/dL for patients undergoing orthopedic surgery, while taking into account individual symptoms and comorbidities [67]. Although heterogeneity was observed in pooled outcomes, mostly ranging from moderate to substantial levels, the consistent direction of effect across subgroup and sensitivity analyses supports the reliability of the observed treatment benefit and indicates that intravenous TXA provides clinically meaningful reductions in blood loss and transfusion risk in patients with extracapsular proximal femur fractures. The variability in the magnitude of the effect likely reflects differences in transfusion practices, patient characteristics and TXA administration protocols, rather than present inconsistency in treatment efficacy. While the consistency in direction of effect supports the clinical benefit of TXA, variability in effect magnitude across studies suggests that results should be interpreted in the context of differences in clinical protocols and healthcare settings. Although the conducted subgroup analyses and sensitivity analyses addressed different methodological approaches in this meta-analysis, in addition to different characteristics of included studies, substantial heterogeneity remained across several reported outcomes, indicating that the pooled estimates should be interpreted cautiously.

The findings of this meta-analysis were consistent, yet somewhat more pronounced regarding the magnitude of benefit with the use of TXA, compared with a previous meta-analysis [68], which included a smaller number of trials and considered geriatric population specifically. It should be noted that comparison with pooled results of previous meta-analyses was difficult due to these combining different study types (RCT and cohort), different types of fractures (intracapsular and extracapsular) and different administration routes (intravenous and topical), but rarely providing comprehensive subgroup analyses that would enable discerning specific clinical circumstances especially by route of administration and type of fracture and consequently type of surgical procedure [26,27,28,29,30,33,69]. A meta-analysis that assessed subgroups by route of TXA administration found that the decrease in the need for transfusion was more substantial with IV use compared to topical use of TXA (27.2% vs. 12.7%, respectively) [30]. Still, it should be noted that this meta-analysis classified the study by Mohib et al. [53] as topical TXA, similar to [69], although the study states IV administration of TXA, as recognized in Cochrane’s systematic review [70] and other meta-analyses [26,29,33,61]. An important aspect that warrants clarification through primary studies is the timing and dosage of TXA, alongside its safety. The rates of adverse events, including thromboembolic events, reported across individual studies were generally similar between the TXA and control groups. Most studies did not observe an increased risk of deep vein thrombosis, pulmonary embolism, myocardial infarction, stroke or other thrombotic events associated with TXA use. However, the included trials were not powered to detect differences in rare safety outcomes, and the duration of follow-up varied. Therefore, while the data across primary studies does not suggest increased thromboembolic risk with intravenous TXA use in patients with extracapsular proximal femur fractures undergoing surgery, definitive conclusions regarding safety are limited and require larger randomized clinical trials specifically designed to assess adverse outcomes. Additionally, it should be noted that the eligibility criteria for trials most often prohibited inclusion of patients with known coagulation disorders, history of thromboembolic events or use of anticoagulants, which may result in study populations with a lower risk for thromboembolic events. While this may limit generalizability to broader clinical populations, particularly elderly patients with multiple comorbidities, the randomized design ensures valid comparisons, and the similar rates of adverse events observed between TXA and control groups suggest that TXA was not associated with an increased safety risk within these studied populations. Still, extrapolating these findings to higher-risk patient populations requires caution. While the small number of reported adverse events precluded meaningful pooling in this meta-analysis, a study that specifically looked into the safety of TXA administration in high-risk patients undergoing surgery for intertrochanteric fractures found no association of TXA use and increased risk of complications or mortality [71]. One meta-analysis reported no significant difference in TBL reduction, transfusion rate or the number of transfused units between RCTs that involved single and multiple instances of TXA administration, but this analysis included both intra- and extracapsular fractures and did not explore different transfusion triggers [72]. This previous pooled analysis also found that a single pre-operative dose of 15 mg/kg was at least not inferior compared to greater dosages in terms of the rate of thromboembolic events. The optimal regimen of TXA administration in different hip fracture surgeries needs to be established in future trials.

### Strengths and Limitations

This meta-analysis had several strengths. First, this study has the most comprehensive and up-to-date literature search. Next, the research question was specific enough to pertain to specific clinical circumstances of extracapsular fractures of proximal femur, comparable surgical interventions, and IV administration of TXA.

However, this study had several limitations. First, the included primary studies varied in TXA dosing regimens and time of administration, precluding specific conclusions regarding the optimal protocol. Further, primary studies exhibited a heterogeneity in the time points for reporting outcomes, i.e., hemoglobin and hematocrit levels, and while this meta-analysis consistently applied the rule of including the time point with the longest follow-up (identical for intervention and control arm in that study) due to its highest clinical relevance, it must be acknowledged that not choosing an identical time point across all studies may under- or overestimate the magnitude of effect. Also, studies differed in transfusion thresholds. Further, heterogeneity in pooled outcomes was considerable, and despite trying to assess this by conducting subgroup analyses and despite sensitivity analyses showing that the results were robust, this issue should be further investigated in future meta-analyses using meta-regression techniques and by improving the methodological planning and detailed reporting of trials. Additionally, this meta-analysis did not pool estimates of adverse outcomes, e.g., thromboembolic events. Although several included studies reported safety data, the absolute number of adverse events was low across intervention and control groups, and reporting was inconsistent across trials, with substantial variations in duration of follow-up. Consequently, and due to limited sample sizes, the included studies were not adequately powered to detect meaningful differences in adverse outcomes and quantitative synthesis of such data could have produced unreliable estimates and potentially overinterpret sparse data, so these were presented narratively only. Also, the predominance of studies conducted in China may limit generalizability to other healthcare systems or warrant further investigation in other settings. Further, primary studies did not uniformly report on outcomes, which should be improved in planning future clinical trials. Finally, this study was not pre-registered, which may introduce bias. Although the methodology (eligibility criteria, outcomes and analytic methods) was defined prior to data extraction and transparently reported, the lack of registration of protocol for this meta-analysis in a registry may increase the risk of selective reporting and should be addressed in future systematic reviews.

## 5. Conclusions

This meta-analysis showed that administration of TXA in patients with extracapsular proximal femur fractures undergoing surgery resulted in a statistically significant benefit for bleeding outcomes, including reduced TBL and HBL, lower declines in Hb and Hct, reduced risk for transfusion and lower number of transfused units. These findings support consideration of TXA as a part of blood management strategies in this population. Still, the effect of TXA should be considered in the context of the individual patient, institutional transfusion practices and transfusion thresholds. Future high-quality randomized trials are needed to standardize the timing of TXA use and dosing regimens, and these trials need to be designed so as to produce a rigorous and comprehensive assessment of outcomes, particularly related to adverse events.

## Figures and Tables

**Table 1:** Characteristics of included studies.

Author, Year, Ref.	Location	Fracture Type/Fixation Method	Sample Size I/C	Males: Females I/C	Mean Age (Years) I/C	Intervention	Control	Transfusion Criteria	AE I/C
Ekinci, 2022 [7]	Turkey	IT AO 31-A1 and 31-A2/PFN	51/51	26:25/19:32	76.0 ± 18.3/79.8 ± 10.5	15 mg/kg TXA IV before incision	Saline	Hb < 8 g/dL or Hb < 10 g/dL with symptoms of hypovolemia	DVT 3/1 PE 1/1 MI 0/0 CVT 1/2
Nikolaou, 2021 [8]	Greece	IT/IMN	42/59	NR *	NR	15 mg/kg IV before incision	Saline	Hb 9 g/dL^−1^ or 10 g/dL^−1^ for patients at risk	PE 0/1 Death 2/1
Tian, 2018 [9]	China	IT A1, A2, A3/PFNA	50/50	19:31/14:36	77.74 ± 6.53/79.25 ± 6.55	10 mg/kg^−1^ IV 10 min preop and 5 h postop	None stated	Hb < 9 mg/dL	DVT 3/2
Ahmed, 2019 [10]	Pakistan	IT/DHS	60/60	NR *	65.60 ± 8.17/65.87 ± 8.60	15 mg/kg TXA preop	Saline	NR	NR
Owen, 2024 [11]	USA	PT OTA/AO 31-A/Cephalomedullary nail	64/64	19:45/19:45	79.1 ± 13.5/79.69 ± 12.7	1 g IV bolus of TXA + 1 g over the next 8 h	Saline	Hb < 7 g/dL or <8 g/dL with persistent symptoms	DVT 3/1 PE 1/0 MI 0/2 Stroke 0/3 Death 7/6
Haghighi, 2017 [12]	Iran	Proximal femoral shaft/IMN	18:20	14:4/17:3	65.11 ± 4.89/66.15 ± 8.51	15 mg/kg IV TXA 20 min before incision	Saline	NR	NR
Luo, 2024 [13]	China	IT/PFNA	56/56	25:31/17:39	80.59 ± 8.11/83.41 ± 7.91	1.5 g TXA q12 h from postadmission day 1 to 3	Saline	Hb < 70 g/L or Hb 70–100 g/L with symptoms of anemia	IVT 8/9 DVT 5/4 PE 0/0 Death 1/1
Majumdar, 2025 [14]	India	IT/Fixation with DHS or dynamic condylar plate	50/50	NR	NR	2 g IV TXA 2 h before surgery + 2 g of TXA after wound closure	Saline	Hb drop by 4 g% or Hct drop by 28% from baseline or drain collection ≥ 500 mL	NR
Wang, 2023 [15]	China	IT/PFNA	33/33	14:19/11:22	64.1 ± 8.3/65.1 ± 7.4	0.5 g TXA IV 20 min preop + 0.5 g TXA sprayed on wound after suturing	Saline	Hb < 80 g/L	VT 1/2
Zhu, 2024 [16]	China	IT/IMN	25/22/32	15:10/7:15/11:21	77.60 ± 9.96/77.56 ± 11.02/80.05 ± 9.31	1 g TXA IV 30 min before surgery/ 1 g TXA IV 30 min before surgery + 1 g at 3, 6 and 9 h after surgery	No TXA	Hb < 7 g/dL or Hb drop > 4 g/dL postoperatively or serious symptoms of anemia	DVT 6/5/8 PE 0/0/0 CCA 0/0/0
Tengberg, 2016 [17]	Denmark	Unstable trochanteric AO 31-A2–2 to 31-A3/IMN	33/39	7:26/14:25	79.8 ± 11.5/75.0 ± 12.6	1 g TXA IV during draping + postop 24 h infusion of 3 g TXA	Saline	Hb < 9.67 g/dL; 6.0 mmol/L	CI 0/1 DVT 0/1 Death 9/2
Zhou, 2019 [18]	China	Stable or unstable IT/PFNA	50/50	15:35/22:28	75.10 ± 8.27/77.82 ± 6.42	1 g/100 mL TXA IV 15 min prior surgery	None	Hb < 70 g/L	DVT 2/3 PE 0/1 MI 0/1 ICI 1/2
Akram, 2021 [45]	Iran	IT Boyd and Griffin type 1, 2 and 3/DHS	39/39	16:23/15:24	44.20 ± 9.64/45.97 ± 9.03	15 mg/kg TXA IV at anesthesia induction + after 3 h	Saline	Hb < 9 g/dL	No AE
Baruah, 2016 [46]	India	Stable trochanteric AO 31A1 and 31A2.1/DHS plate	30/30	24:6/25:5	57.67 ± 14.46/55.33 ± 15.19	15 mg/kg TXA IV 15 min prior to surgery	Saline	Hb < 8.5 g/dL or Hct < 27%	No TAE recorded
Chen, 2019 [47]	China	Trochanteric/DHS and PFNA	88/88	39:49/37:51	76.8 ± 7.0 77.4 ± 6.8	3 doses of 15 mg/kg TXA 10 min before incision + throughout surgery + 3 h after surgery	Saline	Hb < 7.0 g/dL or Hb < 10 g/dL suspected to have myocardial ischemia or hemorrhagic shock	TE 14/12 (DVT 10/11 PE 2/1 MI 0/0 CVA 2/0) Death 5/3
Wang, 2021 [48]	China	IT/PFNA	33/35/32	10:23/9:26/10:22	75.15 ± 9.36/74.31 ± 7.11/72.25 ± 7.65	1 g TXA IV 30 min before incision/1 g TXA IV 30 min before incision and same at 3 h and at 6 h postop	Saline	Hb < 70 g/L or Hb 70–100 g/L considering age, cardiopulmonary function and severity of anemia	IVT 4/3/2
Qiu, 2023 [49]	China	IT AO/OTA 31-A1, 31-A2, 31-A3/TFNA	25/25	12:13/14:11	74.20 ± 6.86/74.40 ± 5.40	1 g TXA IV 15 min preop	Saline	Hb < 70 g/L	IVT 4/2
Lei, 2017 [50]	China	Stable and unstable IT/PFNA	37/40	5:32/7:33	77.80 ± 9.75/79.18 ± 6.50	1 g TXA IV after anesthesia	Saline	Hb < 90 g/L	DVT 2/1 PE 1/1 MI 0/0 ICI 0/0
Luo, 2019 [51]	China	IT AO/OTA 31-A1 to 31-A3/PFNA	44/46	23:21/20:26	75.1 ± 8.0/76.1 ± 9.3	15 mg/kg TXA IV 15 min before incision and 3 h later	Saline	Hb < 8 g/dL or Hb ≥ 8 g/dL with signs of excess blood loss	DVT 1/1 CI 0/3 Death 0/1
Ma, 2021 [52]	China	IT/IT fracture	63/62	21:42/22:40	78.05 ± 7.62/78.66 ± 6.95	1 g of TXA IV on post-traumatic admission	Saline	Hb < 80 g/L or Hb 80–100 g/L with symptomatic anemia	NR
Mohib, 2015 [53]	Pakistan	IT/IT fracture	50/50	21:29/24:26	69.0 ± 10.0/70 ± 9.4	15 mg/kg TXA IV before surgery + 3 h later	Saline	Hb < 70 g/dL	NR
Zhang, 2020 [54]	China	IT/PFNA	15/15/15	7:8/6:9/8:7	71.2/71.6/73.1	IV 1 g of TXA 15 min before incision/ IV 1 g of TXA 15 min before incision + TXA cocktail ** into medullary cavity + TXA cocktail ** around incision before suturing	Saline	Hb < 70 g/L	DVT 0/0/1
Shah, 2023 [55]	Pakistan	IT AO 31A1.2/31A1.3/DHS	100/100	72:28/70:30	48.16 ± 1.75/48.35 ± 1.60	15 mg/kg TXA IV before anesthesia	Saline	Hb < 9 g/dL	DVT 1/1 PE 1/1 MI 0/1 Stroke 1/0 Death 1/2
Yakel, 2023 [56]	USA	Closed IT or ST/IMN	40/49	11:29/14:35	82.2 ± 10.2/79.2 ± 13.0	1 g TXA IV on hospital arrival	Saline	Hb < 8 g/dL	MI 0/2 Death 2/4
Zhang, 2022 [57]	China	IT AO 31A/PFNA	61/61	28:33/34:27	79.11 ± 11.91/76.07 ± 16.60	2 doses of 1 g of TXA IV: 10 min before incision and 3 h later	Saline	Hb < 70 g/L^−1^ or Hb 70–100 g/L^−1^ with symptoms of anemia	TE 3/1 (DVT 2/1 PE 1/0 MI 0/0 Stroke 0/0) Death 1/2

Ref—reference; I—intervention; C—control; AE—adverse event; IT—intertrochanteric; AO—Arbeitsgemeinschaft für Osteosynthesefragen; PFN—proximal femoral nail; TXA—tranexamic acid; IV—intravenous; Hb—hemoglobin; DVT—deep vein thrombosis; PE—pulmonary embolism; MI—myocardial infarction; CVT—cerebrovascular thrombosis; IMN—intramedullary nailing; NR—not reported; PFNA—proximal femoral nail antirotation; DHS—dynamic hip screw; PT—peritrochanteric; OTA—Orthopaedic Trauma Association; Hct—hematocrit; *—provided for the entire study population; IVT—intramuscular venous thrombosis; TFNA—trochanteric fixation nail advanced; VT—venous thrombosis; CCA—cardiocerebral accident; CI—cerebral infarction; ICI—ischemic cerebral infarction; TAE—thromboembolic adverse event; CVA—cerebrovascular accident; **—TXA cocktail (50 mL of NS, 1 g TXA, 0.1 mg epinephrine, 100 mg ropivacaine); ST—subtrochanteric.

**Table 2:** Results of subgroup analyses.

Outcome	Subgroup	Studies (*n*)	Effect Size (95% CI)	I^2^	*p* *
TBL	In China	11	MD = −256.60 (−315.11 to −198.08)	82%	0.81
	Outside China	8	MD = −272.02 (−382.96 to −161.07)	85%	
HBL	In China	11	MD = −215.39 (−285.67 to −145.10)	94%	0.51
	Outside China	1	MD = −275.50 (−440.07 to −110.93)	N/A	
Change in Hb	In China	10	MD = 0.78 (0.55 to 1.01)	68%	0.05
	Outside China	8	MD = 0.43 (0.14 to 0.71)	69%	
Change in Hct	In China	6	MD = 4.12 (1.54 to 6.70)	95%	0.90
	Outside China	3	MD = 4.46 (−0.17 to 9.09)	95%	
Transfusion need	In China	13	RR = 0.48 (0.35 to 0.66)	62%	0.13
	Outside China	10	RR = 0.66 (0.50 to 0.89)	82%	
Transfusion need	‘Restrictive’ threshold	15	RR = 0.47 (0.35 to 0.63)	56%	0.04
	‘Liberal’ threshold	6	RR = 0.72 (0.54 to 0.96)	85%	

CI—confidence interval; TBL—total blood loss; MD—mean difference; N/A—not available; HBL—hidden blood loss; Hb—hemoglobin; Hct—hematocrit, RR—risk ratio. *—*p* for subgroup differences.

## Data Availability

The original contributions presented in this study are included in the article/supplementary material. Further inquiries can be directed to the corresponding author.

## Supplementary Materials

The following supporting information can be downloaded at: https://www.mdpi.com/article/10.3390/pharmaceutics18030374/s1, Table S1. PRISMA Checklist. Table S2. Search strategies. Figure S1. Forest plot for the effect of tranexamic acid (TXA) versus control on change in hemoglobin with correlation coefficient r (A) 0.3 and (B) 0.7. Figure S2. Forest plot for the effect of tranexamic acid (TXA) versus control on change in hematocrit with correlation coefficient r (A) 0.3 and (B) 0.7. Figure S3. Forest plot for the effect of tranexamic acid (TXA) versus control on (A) Total blood loss and (B) Hidden blood loss, considering trial’s arm with multiple doses of TXA in multi-arm trials. Figure S4. Forest plot for the effect of tranexamic acid (TXA) versus control on change in (A) hemoglobin and (B) hematocrit, considering trial’s arm with multiple doses of TXA in multi-arm trials. Figure S5. Forest plot for the effect of tranexamic acid (TXA) versus control on (A) transfusion rate and (B) number of transfused units per patient, considering trial’s arm with multiple doses of TXA in multi-arm trials. Figure S6. Risk of bias assessment for total blood loss and hidden blood loss (A) traffic light plot and (B) summary plot. Figure S7. Risk of bias assessment for change in hemoglobin and hematocrit (A) traffic light plot and (B) summary plot. Figure S8. Risk of bias assessment for transfusion rate (A) traffic light plot and (B) summary plot. Figure S9. Risk of bias assessment for number of transfused units per patient (A) traffic light plot and (B) summary plot. Figure S10. Funnel plots for estimates in meta-analysis of tranexamic acid use and (A) total blood loss, (B) hidden blood loss, (C) change in hemoglobin, (D) change in hematocrit, (E) risk for transfusion and (F) number of transfused units per patient.
