# Supplementary material for: Tranexamic Acid for Reduction of Blood Loss in Patients with Extracapsular Proximal Femur Fractures: Systematic Review and Meta-Analysis of Randomized Clinical Trials
Source: Pharmaceutics. 2026 Mar 18;18(3):374. doi: 10.3390/pharmaceutics18030374 (PMC13029286; doi:10.3390/pharmaceutics18030374)
Supplement: Supplementary file 1 [file pharmaceutics-18-00374-s001.zip › pharmaceutics-4198265-supplementary.pdf]

Table S1. PRISMA Checklist.			
**Section and Topic**	**Item #**	**Checklist item**	**Location where item is reported**
**TITLE**			
Title	1	Identify the report as a systematic review.	Page 1
**ABSTRACT**			
Abstract	2	See the PRISMA 2020 for Abstracts checklist.	Page 1
**INTRODUCTION**			
Rationale	3	Describe the rationale for the review in the context of existing knowledge.	Page 2
Objectives	4	Provide an explicit statement of the objective(s) or question(s) the review addresses.	Page 2
**METHODS**			
Eligibility criteria	5	Specify the inclusion and exclusion criteria for the review and how studies were grouped for the syntheses.	Page 3
Information sources	6	Specify all databases, registers, websites, organisations, reference lists and other sources searched or consulted to identify studies. Specify the date when each source was last searched or consulted.	Page 3
Search strategy	7	Present the full search strategies for all databases, registers and websites, including any filters and limits used.	Page 3, Table S2
Selection process	8	Specify the methods used to decide whether a study met the inclusion criteria of the review, including how many reviewers screened each record and each report retrieved, whether they worked independently, and if applicable, details of automation tools used in the process.	Page 3
Data collection process	9	Specify the methods used to collect data from reports, including how many reviewers collected data from each report, whether they worked independently, any processes for obtaining or confirming data from study investigators, and if applicable, details of automation tools used in the process.	Page 3
Data items	10a	List and define all outcomes for which data were sought. Specify whether all results that were compatible with each outcome domain in each study were sought (e.g. for all measures, time points, analyses), and if not, the methods used to decide which results to collect.	Pages 3-4
	10b	List and define all other variables for which data were sought (e.g. participant and intervention characteristics, funding sources). Describe any assumptions made about any missing or unclear information.	Pages 3-4
Study risk of bias assessment	11	Specify the methods used to assess risk of bias in the included studies, including details of the tool(s) used, how many reviewers assessed each study and whether they worked independently, and if applicable, details of automation tools used in the process.	Pages 3-4
Effect measures	12	Specify for each outcome the effect measure(s) (e.g. risk ratio, mean difference) used in the synthesis or presentation of results.	Page 4

Table S1. PRISMA Checklist.

Section and Topic	Item #	Checklist item	Location where item is reported
Synthesis methods	13a	Describe the processes used to decide which studies were eligible for each synthesis (e.g. tabulating the study intervention characteristics and comparing against the planned groups for each synthesis (item #5)).	Page 4
	13b	Describe any methods required to prepare the data for presentation or synthesis, such as handling of missing summary statistics, or data conversions.	Page 4
	13c	Describe any methods used to tabulate or visually display results of individual studies and syntheses.	Page 4
	13d	Describe any methods used to synthesize results and provide a rationale for the choice(s). If meta-analysis was performed, describe the model(s), method(s) to identify the presence and extent of statistical heterogeneity, and software package(s) used.	Page 4
	13e	Describe any methods used to explore possible causes of heterogeneity among study results (e.g. subgroup analysis, meta-regression).	Page 4
	13f	Describe any sensitivity analyses conducted to assess robustness of the synthesized results.	Page 4
Reporting bias assessment	14	Describe any methods used to assess risk of bias due to missing results in a synthesis (arising from reporting biases).	Page 4
Certainty assessment	15	Describe any methods used to assess certainty (or confidence) in the body of evidence for an outcome.	Page 4
**RESULTS**			
Study selection	16a	Describe the results of the search and selection process, from the number of records identified in the search to the number of studies included in the review, ideally using a flow diagram.	Pages 5-6, Figure 1
	16b	Cite studies that might appear to meet the inclusion criteria, but which were excluded, and explain why they were excluded.	Figure 1
Study characteristics	17	Cite each included study and present its characteristics.	Pages 5-10, Table 1
Risk of bias in studies	18	Present assessments of risk of bias for each included study.	Page 11, Figures S6-S9
Results of individual studies	19	For all outcomes, present, for each study: (a) summary statistics for each group (where appropriate) and (b) an effect estimate and its precision (e.g. confidence/credible interval), ideally using structured tables or plots.	Pages 11-14
Results of syntheses	20a	For each synthesis, briefly summarise the characteristics and risk of bias among contributing studies.	Pages 10-14

Table S1. PRISMA Checklist.			
**Section and Topic**	**Item #**	**Checklist item**	**Location where item is reported**
	20b	Present results of all statistical syntheses conducted. If meta-analysis was done, present for each the summary estimate and its precision (e.g. confidence/credible interval) and measures of statistical heterogeneity. If comparing groups, describe the direction of the effect.	Pages 10-13
	20c	Present results of all investigations of possible causes of heterogeneity among study results.	Page 13
	20d	Present results of all sensitivity analyses conducted to assess the robustness of the synthesized results.	Page 13
Reporting biases	21	Present assessments of risk of bias due to missing results (arising from reporting biases) for each synthesis assessed.	Pages 11-14
Certainty of evidence	22	Present assessments of certainty (or confidence) in the body of evidence for each outcome assessed.	Pages 11-14,<br>Figures S1-S5
**DISCUSSION**			
Discussion	23a	Provide a general interpretation of the results in the context of other evidence.	Pages 14-18
	23b	Discuss any limitations of the evidence included in the review.	Pages 15-18
	23c	Discuss any limitations of the review processes used.	Page 18
	23d	Discuss implications of the results for practice, policy, and future research.	Pages 14-18
**OTHER INFORMATION**			
Registration and protocol	24a	Provide registration information for the review, including register name and registration number, or state that the review was not registered.	Page 3
	24b	Indicate where the review protocol can be accessed, or state that a protocol was not prepared.	Page 3
	24c	Describe and explain any amendments to information provided at registration or in the protocol.	/
Support	25	Describe sources of financial or non-financial support for the review, and the role of the funders or sponsors in the review.	Page 19
Competing interests	26	Declare any competing interests of review authors.	Page 19

Table S1. PRISMA Checklist.			
**Section and Topic**	**Item #**	**Checklist item**	**Location where item is reported**
Availability of data, code and other materials	27	Report which of the following are publicly available and where they can be found: template data collection forms; data extracted from included studies; data used for all analyses; analytic code; any other materials used in the review.	Page 23

Table S2. Search strategy

Database	Search strategy
PubMed	(("Tranexamic Acid"[Mesh] OR "tranexamic acid"[tw] OR TXA[tw] OR "trans-4-(aminomethyl)cyclohexanecarboxylic acid" [tw]) AND ("Hip Fractures"[Mesh] OR "Femoral Fractures"[Mesh] OR "hip fracture*"[tw] OR "intertrochanteric fracture*"[tw] OR "subtrochanteric fracture*"[tw] OR "pertrochanteric fracture*"[tw] OR "femoral neck fracture*"[tw] OR "proximal femur fracture*"[tw])) AND ("Hemorrhage"[Mesh] OR bleeding[tw] OR hemorrhage[tw] OR haemorrhage[tw] OR "blood loss"[tw])
Scopus	(INDEXTERMS ("Tranexamic Acid") OR TITLE-ABS-KEY ("tranexamic acid") OR TITLE-ABS-KEY ("TXA") OR TITLE-ABS-KEY ("trans-4-(aminomethyl)cyclohexanecarboxylic acid")) AND (INDEXTERMS ("Hip Fractures") OR INDEXTERMS ("Femoral Fractures") OR TITLE-ABS-KEY ("hip fracture*") OR TITLE-ABS-KEY ("intertrochanteric fracture*") OR TITLE-ABS-KEY ("subtrochanteric fracture*") OR TITLE-ABS-KEY ("pertrochanteric fracture*") OR TITLE-ABS-KEY ("femoral neck fracture*") OR TITLE-ABS-KEY ("proximal femur fracture*")) AND (INDEXTERMS ("Hemorrhage") OR TITLE-ABS-KEY ("bleeding") OR TITLE-ABS-KEY ("hemorrhage") OR TITLE-ABS-KEY ("haemorrhage") OR TITLE-ABS-KEY ("blood loss"))
Web of Science	(("Tranexamic Acid" OR "tranexamic acid" OR TXA OR "trans-4-(aminomethyl)cyclohexanecarboxylic acid") AND ("Hip Fractures" OR "Femoral Fractures" OR "hip fracture*" OR "intertrochanteric fracture*" OR "subtrochanteric fracture*" OR "pertrochanteric fracture*" OR "femoral neck fracture*" OR "proximal femur fracture*")) AND (Hemorrhage OR bleeding OR hemorrhage OR haemorrhage OR "blood loss")
Cochrane Central	([mh "Tranexamic Acid"] OR "tranexamic acid":ti,ab,kw OR TXA:ti,ab,kw OR "trans-4-(aminomethyl)cyclohexanecarboxylic acid":ti,ab,kw) AND ([mh "Hip Fractures"] OR [mh "Femoral Fractures"] OR ("hip" NEXT fracture*):ti,ab,kw OR ("intertrochanteric" NEXT fracture*):ti,ab,kw OR ("subtrochanteric" NEXT fracture*):ti,ab,kw OR ("pertrochanteric" NEXT fracture*):ti,ab,kw OR ("femoral neck" NEXT fracture*):ti,ab,kw OR ("proximal femur" NEXT fracture*):ti,ab,kw) AND ([mh "Hemorrhage"] OR bleeding:ti,ab,kw OR hemorrhage:ti,ab,kw OR haemorrhage:ti,ab,kw OR "blood loss":ti,ab,kw)

(A)

Study or Subgroup	TXA Mean	TXA SD	TXA Total	Control Mean	Control SD	Control Total	Weight	Mean Difference IV, Random, 95% CI
Ahmed 2019	0.28	1.045	60	0.04	1.131	60	6.7%	0.24 [-0.15, 0.63]
Akram 2021	-0.947	0.1054	39	-1.036	0.09402	39	7.9%	0.09 [0.04, 0.13]
Baruah 2016	-0.38	1.621	30	-1.1	1.705	30	4.3%	0.72 [-0.12, 1.56]
Chen 2019	-2.18	1.284	88	-2.63	1.297	88	6.7%	0.45 [0.07, 0.83]
Ekinci 2022	-1.6	1.85	51	-2.1	1.657	51	5.1%	0.50 [-0.18, 1.18]
Haghighi 2017	-1.75	0.74	18	-2.04	1.97	20	3.9%	0.29 [-0.64, 1.22]
Lei 2017	-0.845	1.6164	37	-1.185	1.5306	40	5.0%	0.34 [-0.36, 1.04]
Luo 2019	-0.8	2.018	44	-1.4	1.85	46	4.5%	0.60 [-0.20, 1.40]
Luo 2024	-1.841	0.756	56	-2.575	0.945	56	7.1%	0.73 [0.42, 1.05]
Ma 2021	-1.55	0.7121	63	-2.776	0.8309	62	7.3%	1.23 [0.95, 1.50]
Majumdar 2025	-0.94	1.215	50	-1.73	1.154	50	6.3%	0.79 [0.33, 1.25]
Mohib 2015	-1.3	2.531	50	-2.4	2.49	50	3.7%	1.10 [0.12, 2.08]
Qiu 2023	-3.652	0.692	25	-4.156	0.758	25	6.6%	0.50 [0.10, 0.91]
Shah 2023	-1.1	4.115	100	-1.2	2.362	100	3.9%	0.10 [-0.83, 1.03]
Wang 2023	-2.791	1.4705	33	-4.182	1.488	33	4.9%	1.39 [0.68, 2.10]
Zhang 2022	-1.359	0.568	61	-2.031	0.814	61	7.4%	0.67 [0.42, 0.92]
Zhou 2019	-1.481	2.7577	50	-2.739	3.5606	50	2.8%	1.26 [0.01, 2.51]
Zhu 2024	-2.076	0.889	25	-3.078	1.053	32	6.1%	1.00 [0.50, 1.51]
**Total (95% CI)**			**880**			**893**	**100.0%**	**0.65 [0.39, 0.90]**

Heterogeneity: Tau² = 0.22; Chi² = 142.30, df = 17 (P < 0.00001); I² = 88%
Test for overall effect: Z = 4.92 (P < 0.00001)

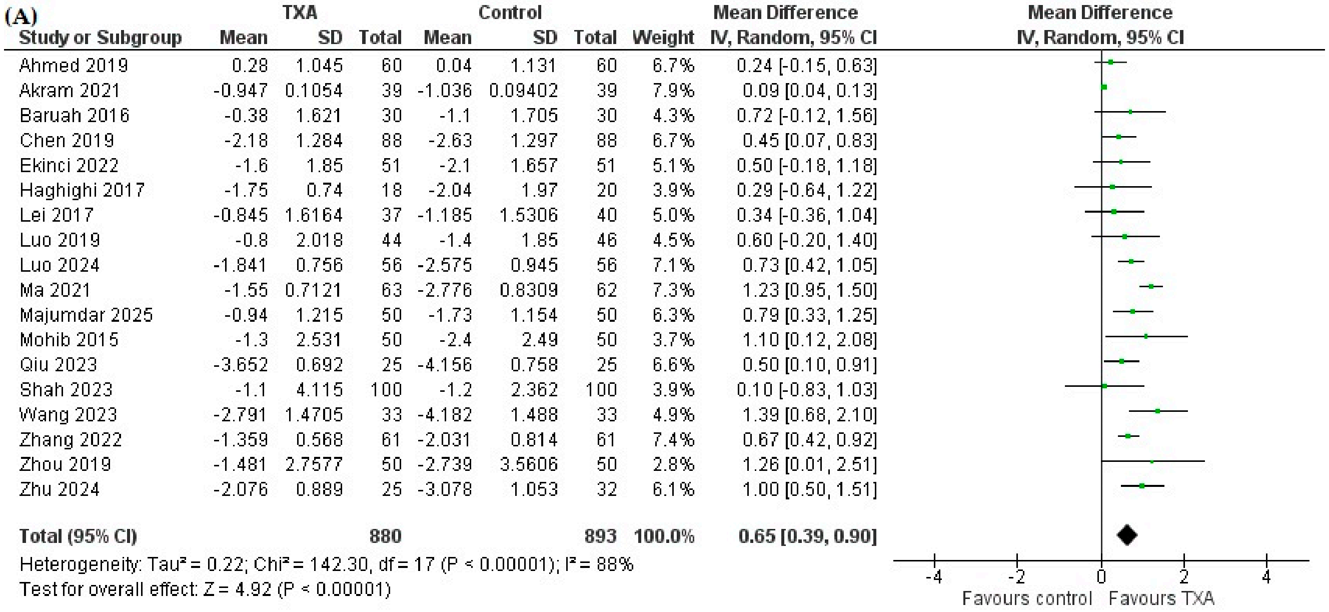

(B)

Study or Subgroup	TXA Mean	TXA SD	TXA Total	Control Mean	Control SD	Control Total	Weight	Mean Difference IV, Random, 95% CI
Ahmed 2019	0.28	0.697	60	0.04	0.827	60	6.4%	0.24 [-0.03, 0.51]
Akram 2021	-0.947	0.1054	39	-1.036	0.09402	39	6.9%	0.09 [0.04, 0.13]
Baruah 2016	-0.38	1.061	30	-1.1	1.133	30	5.2%	0.72 [0.16, 1.28]
Chen 2019	-2.18	1.044	88	-2.63	1.038	88	6.3%	0.45 [0.14, 0.76]
Ekinci 2022	-1.6	1.232	51	-2.1	1.084	51	5.7%	0.50 [0.05, 0.95]
Haghighi 2017	-1.75	0.74	18	-2.04	1.97	20	3.6%	0.29 [-0.64, 1.22]
Lei 2017	-0.845	1.0848	37	-1.185	1.0037	40	5.6%	0.34 [-0.13, 0.81]
Luo 2019	-0.8	1.33	44	-1.4	1.232	46	5.3%	0.60 [0.07, 1.13]
Luo 2024	-1.841	0.756	56	-2.575	0.945	56	6.2%	0.73 [0.42, 1.05]
Ma 2021	-1.55	0.4983	63	-2.776	0.5444	62	6.7%	1.23 [1.04, 1.41]
Majumdar 2025	-0.94	0.798	50	-1.73	0.755	50	6.3%	0.79 [0.49, 1.09]
Mohib 2015	-1.3	1.718	50	-2.4	1.714	50	4.6%	1.10 [0.43, 1.77]
Qiu 2023	-3.652	0.4894	25	-4.156	0.5097	25	6.4%	0.50 [0.23, 0.78]
Shah 2023	-1.1	3.089	100	-1.2	1.756	100	4.5%	0.10 [-0.60, 0.80]
Wang 2023	-2.791	1.2074	33	-4.182	1.3859	33	4.8%	1.39 [0.76, 2.02]
Zhang 2022	-1.359	0.568	61	-2.031	0.814	61	6.5%	0.67 [0.42, 0.92]
Zhou 2019	-1.481	1.8546	50	-2.739	2.6877	50	3.7%	1.26 [0.35, 2.16]
Zhu 2024	-2.076	0.889	25	-3.078	1.053	32	5.4%	1.00 [0.50, 1.51]
**Total (95% CI)**			**880**			**893**	**100.0%**	**0.65 [0.40, 0.90]**

Heterogeneity: Tau² = 0.24; Chi² = 233.18, df = 17 (P < 0.00001); I² = 93%
Test for overall effect: Z = 5.08 (P < 0.00001)

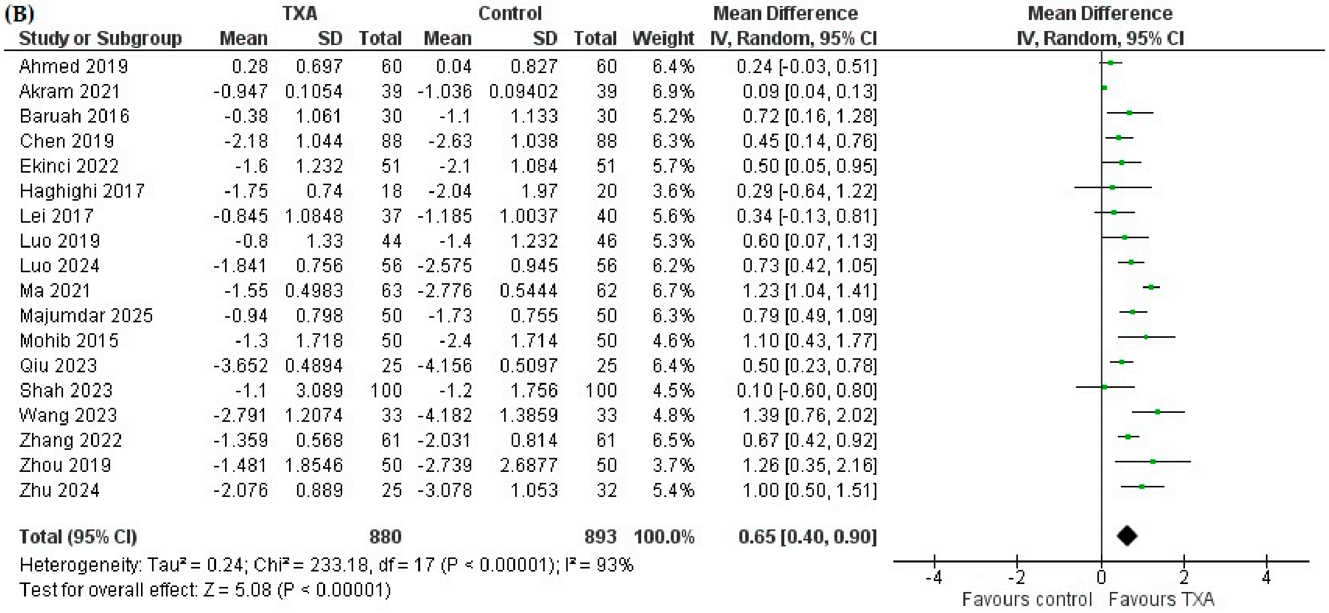

Supplementary Figure S1. Forest plot for the effect of tranexamic acid (TXA) versus control on change in hemoglobin with correlation coefficient r (A) 0.3 and (B) 0.7.

(A)

Study or Subgroup	TXA Mean	TXA SD	TXA Total	Control Mean	Control SD	Control Total	Weight	Mean Difference IV, Random, 95% CI
Ahmed 2019	-3.9	9.009	60	-13.92	4.031	60	10.4%	10.02 [7.52, 12.52]
Baruah 2016	-1.03	2.738	30	-2.6	2.62	30	11.6%	1.57 [0.21, 2.93]
Ekinci 2022	-5	5.246	51	-7	4.851	51	11.0%	2.00 [0.04, 3.96]
Lei 2017	-2.4	4.523	37	-4.45	5.97	40	10.6%	2.05 [-0.31, 4.41]
Ma 2021	-10.17	3.514	63	-16.27	2.868	62	11.7%	6.10 [4.98, 7.22]
Qiu 2023	-7.01	3.25	25	-10.8	2.962	25	11.3%	3.79 [2.07, 5.51]
Wang 2023	-7.12	2.991	33	-16.64	2.989	33	11.5%	9.52 [8.08, 10.96]
Zhou 2019	-3.69	5.604	50	-4.53	6.848	50	10.5%	0.84 [-1.61, 3.29]
Zhu 2024	-7.04	2.99	25	-9.03	2.94	32	11.4%	1.99 [0.44, 3.54]
**Total (95% CI)**			**374**			**383**	**100.0%**	**4.22 [2.03, 6.40]**

Heterogeneity: Tau² = 10.26; Chi² = 120.64, df = 8 (P < 0.00001); I² = 93%
Test for overall effect: Z = 3.78 (P = 0.0002)

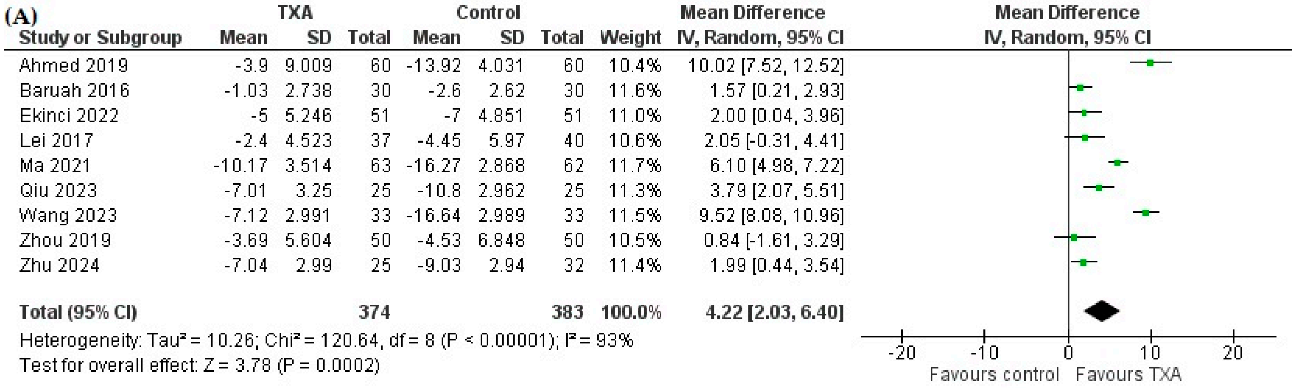

(B)

Study or Subgroup	TXA Mean	TXA SD	TXA Total	Control Mean	Control SD	Control Total	Weight	Mean Difference IV, Random, 95% CI
Ahmed 2019	-3.9	5.967	60	-13.92	2.808	60	10.9%	10.02 [8.35, 11.69]
Baruah 2016	-1.03	2.76	30	-2.6	1.799	30	11.2%	1.57 [0.39, 2.75]
Ekinci 2022	-5	3.487	51	-7	3.176	51	11.2%	2.00 [0.71, 3.29]
Lei 2017	-2.4	3.065	37	-4.45	4.201	40	10.9%	2.05 [0.42, 3.68]
Ma 2021	-10.17	2.487	63	-16.27	1.953	62	11.5%	6.10 [5.32, 6.88]
Qiu 2023	-7.01	2.131	25	-10.8	1.983	25	11.3%	3.79 [2.65, 4.93]
Wang 2023	-7.12	2.278	33	-16.64	2.022	33	11.3%	9.52 [8.48, 10.56]
Zhou 2019	-3.69	3.926	50	-4.53	4.99	50	10.8%	0.84 [-0.92, 2.60]
Zhu 2024	-7.04	2.99	25	-9.03	2.94	32	11.0%	1.99 [0.44, 3.54]
**Total (95% CI)**			**374**			**383**	**100.0%**	**4.23 [2.05, 6.40]**

Heterogeneity: Tau² = 10.60; Chi² = 220.14, df = 8 (P < 0.00001); I² = 96%
Test for overall effect: Z = 3.81 (P = 0.0001)

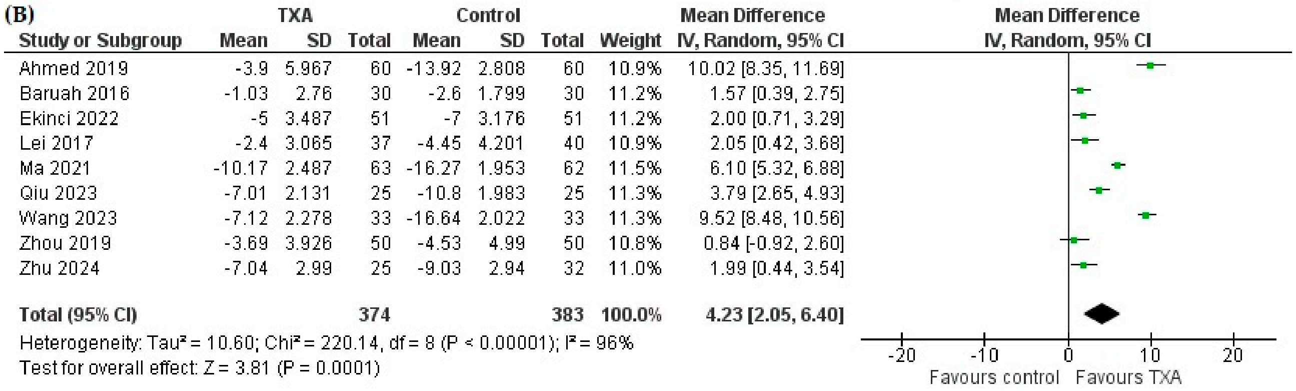

Supplementary Figure S2. Forest plot for the effect of tranexamic acid (TXA) versus control on change in hematocrit with correlation coefficient r (A) 0.3 and (B) 0.7.

(A)

Study or Subgroup	Favours TXA Mean	SD	Total	Control Mean	SD	Total	Weight	Mean Difference IV, Random, 95% CI
Ahmed 2019	395.78	204	60	427.8	146.9	60	7.1%	-32.02 [-95.63, 31.59]
Baruah 2016	408.97	106.35	30	676.67	87.88	30	7.4%	-267.70 [-317.07, -218.33]
Chen 2019	411	108.8	88	616.5	106.3	88	7.7%	-205.50 [-237.28, -173.72]
Ekinci 2022	684.6	370.1	51	971.2	505.3	51	4.4%	-286.60 [-458.50, -114.70]
Lei 2017	279.35	209.11	37	417.89	289.56	40	5.9%	-138.54 [-250.75, -26.33]
Luo 2019	384.5	366.3	44	566.2	361.5	46	4.9%	-181.70 [-332.12, -31.28]
Majumdar 2025	342	156	50	605	179	50	7.1%	-263.00 [-328.81, -197.19]
Nikolaou 2021	844	596.425	38	1,269.1	924.85	46	2.0%	-425.10 [-752.80, -97.40]
Owen 2024	1,593	1,215	64	2,013	1,402	64	1.2%	-420.00 [-874.52, 34.52]
Qiu 2023	712.13	128.63	25	1,232.36	221.22	25	6.2%	-520.23 [-620.54, -419.92]
Tengberg 2016	1,529.6	1,012.7	33	2,100.4	115.6	39	1.8%	-570.80 [-918.22, -223.38]
Tian 2018	515.3	278.79	50	696.58	275	50	6.0%	-181.28 [-289.82, -72.74]
Wang 2021	499.2	189.8	35	859.41	165.03	32	6.6%	-360.21 [-445.20, -275.22]
Wang 2023	664.27	90.23	33	959.34	69.54	33	7.6%	-295.07 [-333.94, -256.20]
Yakel 2023	1,181.8	537	30	1,548.4	636.8	38	2.5%	-366.60 [-645.74, -87.46]
Zhang 2020	434.93	66.84	15	770.53	149.34	15	6.7%	-335.60 [-418.40, -252.80]
Zhang 2022	712.11	429.71	61	1,103.55	412.04	61	4.9%	-391.44 [-540.84, -242.04]
Zhou 2019	563.37	197.51	50	819.25	273.96	50	6.4%	-255.88 [-349.49, -162.27]
Zhu 2024	640.86	337.22	22	1,226.27	458.22	32	3.5%	-585.41 [-797.69, -373.13]
**Total (95% CI)**			**816**			**850**	**100.0%**	**-287.60 [-341.30, -233.91]**

Heterogeneity: Tau² = 9464.88; Chi² = 121.82, df = 18 (P < 0.00001); I² = 85%
Test for overall effect: Z = 10.50 (P < 0.00001)

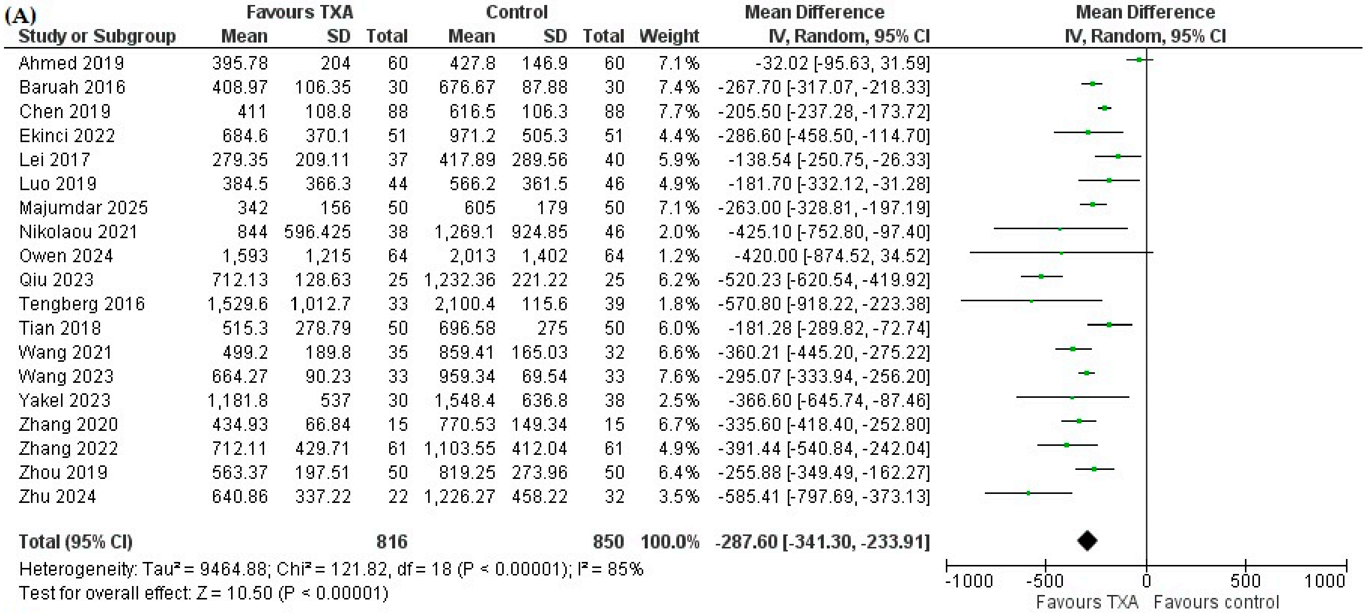

(B)

Study or Subgroup	Favours TXA Mean	SD	Total	Control Mean	SD	Total	Weight	Mean Difference IV, Random, 95% CI
Chen 2019	266	56.5	88	342.5	81	88	10.1%	-76.50 [-97.13, -55.87]
Ekinci 2022	582.3	341.2	51	857.8	493.1	51	6.6%	-275.50 [-440.07, -110.93]
Lei 2017	210.09	202.14	37	359.35	290.12	40	8.2%	-149.26 [-260.28, -38.24]
Luo 2024	515.52	224.9	56	681.75	291.74	56	8.6%	-166.23 [-262.71, -69.75]
Ma 2021	644.6	54.4	63	879.2	50.2	62	10.2%	-234.60 [-252.95, -216.25]
Qiu 2023	458.53	120.83	25	973.16	260.9	25	8.2%	-514.63 [-627.34, -401.92]
Tian 2018	401.96	260.72	50	532.6	253.41	50	8.5%	-130.64 [-231.42, -29.86]
Wang 2021	359.03	208.57	35	719.22	204.08	32	8.6%	-360.19 [-459.05, -261.33]
Zhang 2020	337.6	61.34	15	624	140.21	15	9.1%	-286.40 [-363.85, -208.95]
Zhang 2022	640.96	421.63	61	1,010.11	398.96	61	7.2%	-369.15 [-514.82, -223.48]
Zhou 2019	410.42	178.23	50	571.19	218.13	50	9.1%	-160.77 [-238.85, -82.69]
Zhu 2024	583.13	318.08	22	1,154.39	452.06	32	5.6%	-571.26 [-776.68, -365.84]
**Total (95% CI)**			**553**			**562**	**100.0%**	**-259.55 [-331.30, -187.80]**

Heterogeneity: Tau² = 13111.66; Chi² = 207.74, df = 11 (P < 0.00001); I² = 95%
Test for overall effect: Z = 7.09 (P < 0.00001)

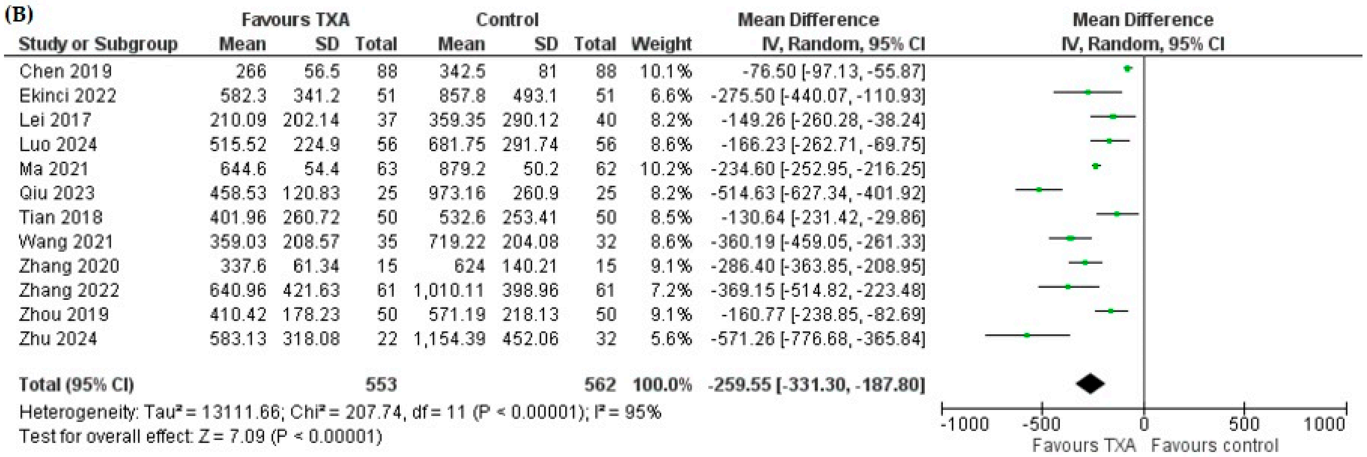

Supplementary Figure S3. Forest plot for the effect of tranexamic acid (TXA) versus control on (A) Total blood loss and (B) Hidden blood loss, considering trial's arm with multiple doses of TXA in multi-arm trials.

(A)

Study or Subgroup	TXA Mean	TXA SD	TXA Total	Control Mean	Control SD	Control Total	Weight	Mean Difference IV, Random, 95% CI
Ahmed 2019	0.28	0.889	60	0.04	0.991	60	6.5%	0.24 [-0.10, 0.58]
Akram 2021	-0.947	0.1054	39	-1.036	0.09402	39	7.3%	0.09 [0.04, 0.13]
Baruah 2016	-0.38	1.37	30	-1.1	1.448	30	4.8%	0.72 [0.01, 1.43]
Chen 2019	-2.18	1.17	88	-2.63	1.175	88	6.5%	0.45 [0.10, 0.80]
Ekinci 2022	-1.6	1.572	51	-2.1	1.4	51	5.4%	0.50 [-0.08, 1.08]
Haghighi 2017	-1.75	0.74	18	-2.04	1.97	20	3.8%	0.29 [-0.64, 1.22]
Lei 2017	-0.845	1.3765	37	-1.185	1.2942	40	5.3%	0.34 [-0.26, 0.94]
Luo 2019	-0.8	1.709	44	-1.4	1.572	46	4.9%	0.60 [-0.08, 1.28]
Luo 2024	-1.841	0.756	56	-2.575	0.945	56	6.6%	0.73 [0.42, 1.05]
Ma 2021	-1.55	0.6146	63	-2.776	0.7024	62	6.9%	1.23 [0.99, 1.46]
Majumdar 2025	-0.94	1.028	50	-1.73	0.975	50	6.3%	0.79 [0.40, 1.18]
Mohib 2015	-1.3	2.163	50	-2.4	2.138	50	4.2%	1.10 [0.26, 1.94]
Qiu 2023	-3.652	0.593	25	-4.156	0.6459	25	6.5%	0.50 [0.16, 0.85]
Shah 2023	-1.1	3.639	100	-1.2	2.081	100	4.3%	0.10 [-0.72, 0.92]
Wang 2023	-2.791	1.3454	33	-4.182	1.4378	33	5.0%	1.39 [0.72, 2.06]
Zhang 2022	-1.359	0.568	61	-2.031	0.814	61	6.8%	0.67 [0.42, 0.92]
Zhou 2019	-1.481	2.35	50	-2.739	3.1545	50	3.3%	1.26 [0.17, 2.35]
Zhu 2024	-1.564	0.863	22	-3.078	1.053	32	5.7%	1.51 [1.00, 2.03]
**Total (95% CI)**			**877**			**893**	**100.0%**	**0.68 [0.41, 0.94]**

Heterogeneity: Tau² = 0.25; Chi² = 187.90, df = 17 (P < 0.00001); I² = 91%
Test for overall effect: Z = 5.03 (P < 0.00001)

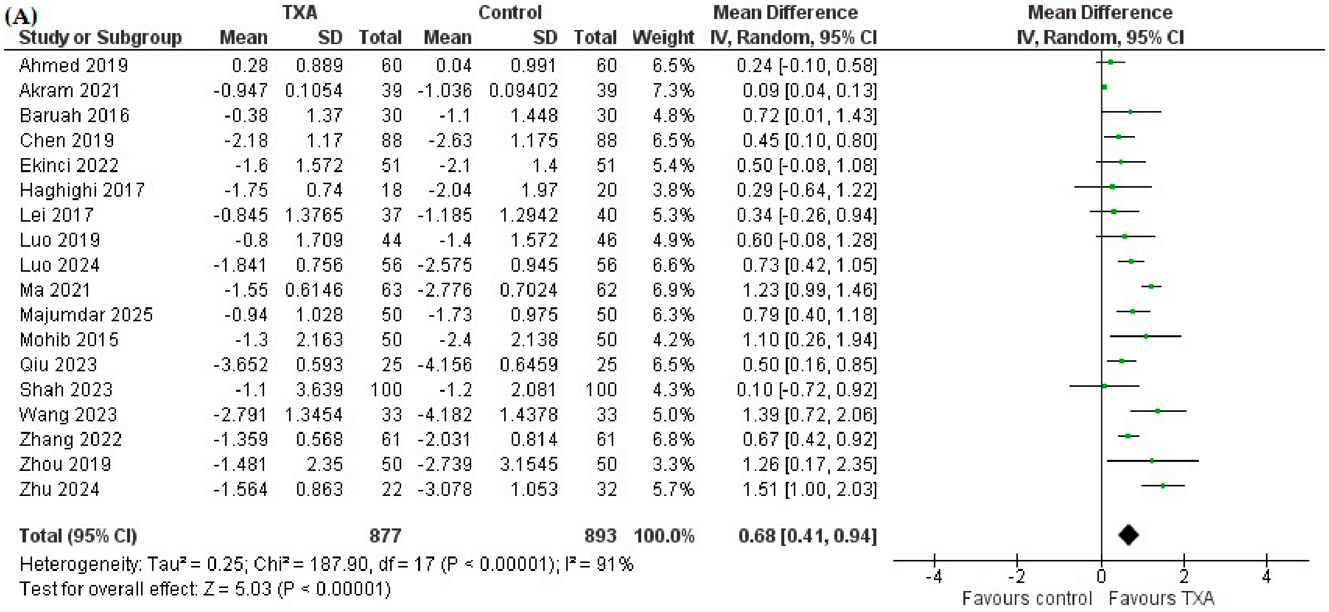

(B)

Study or Subgroup	TXA Mean	TXA SD	TXA Total	Control Mean	Control SD	Control Total	Weight	Mean Difference IV, Random, 95% CI
Ahmed 2019	-3.9	7.641	60	-13.92	3.474	60	10.6%	10.02 [7.90, 12.14]
Baruah 2016	-1.03	2.316	30	-2.6	2.45	30	11.4%	1.57 [0.36, 2.78]
Ekinci 2022	-5	4.454	51	-7	4.1	51	11.1%	2.00 [0.34, 3.66]
Lei 2017	-2.4	3.864	37	-4.45	5.162	40	10.7%	2.05 [0.02, 4.08]
Ma 2021	-10.17	3.044	63	-16.27	2.453	62	11.6%	6.10 [5.13, 7.07]
Qiu 2023	-7.01	2.748	25	-10.8	2.52	25	11.2%	3.79 [2.33, 5.25]
Wang 2023	-7.12	2.659	33	-16.64	2.552	33	11.4%	9.52 [8.26, 10.78]
Zhou 2019	-3.69	4.838	50	-4.53	5.992	50	10.6%	0.84 [-1.29, 2.97]
Zhu 2024	-5.26	1.99	22	-9.03	2.94	32	11.4%	3.77 [2.46, 5.08]
**Total (95% CI)**			**371**			**383**	**100.0%**	**4.42 [2.34, 6.50]**

Heterogeneity: Tau² = 9.42; Chi² = 147.10, df = 8 (P < 0.00001); I² = 95%
Test for overall effect: Z = 4.17 (P < 0.0001)

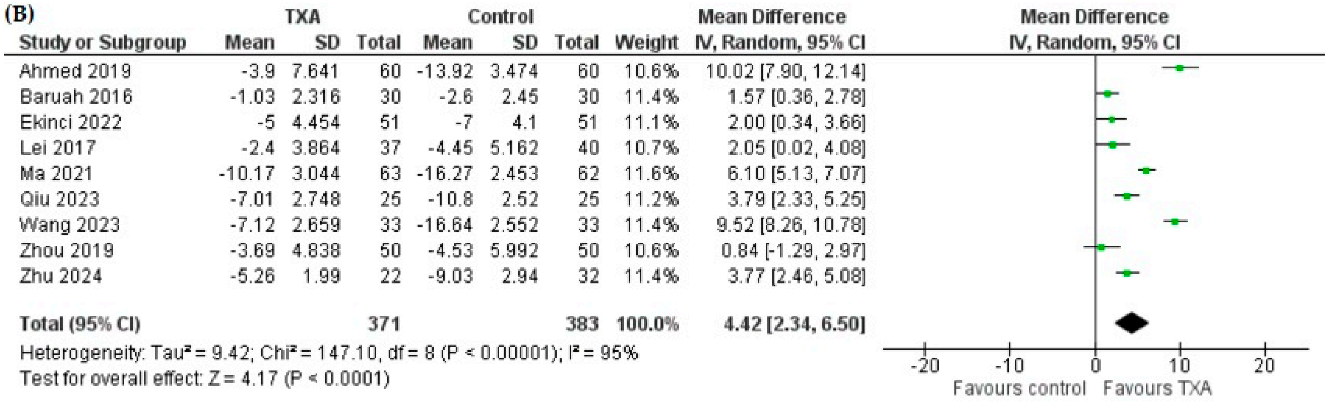

Supplementary Figure S4. Forest plot for the effect of tranexamic acid (TXA) versus control on change in (A) hemoglobin and (B) hematocrit, considering trial's arm with multiple doses of TXA in multi-arm trials.

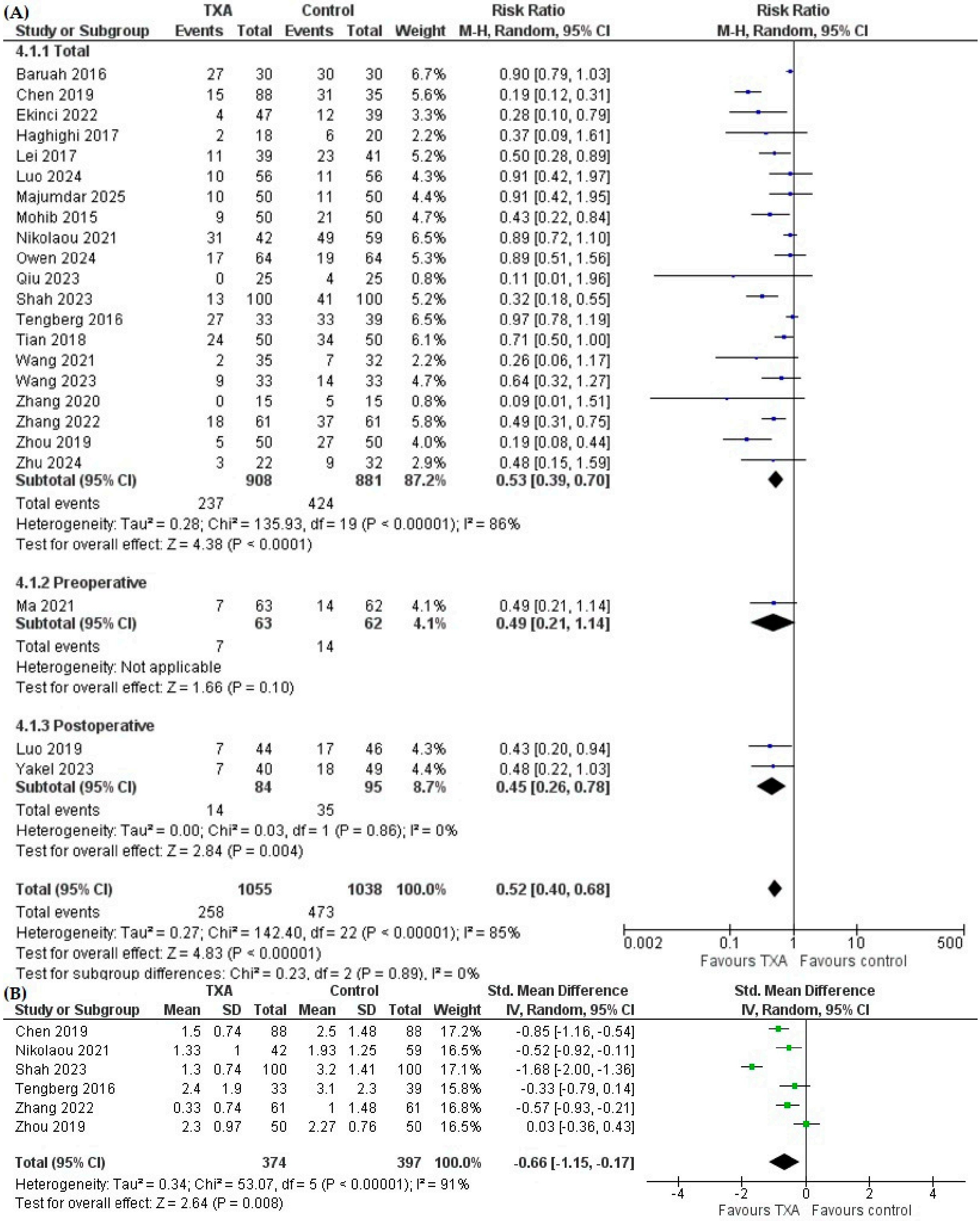

(A)

Study or Subgroup	TXA Events	TXA Total	Control Events	Control Total	Weight	Risk Ratio M-H, Random, 95% CI
**4.1.1 Total**						
Baruah 2016	27	30	30	30	6.7%	0.90 [0.79, 1.03]
Chen 2019	15	88	31	35	5.6%	0.19 [0.12, 0.31]
Ekinci 2022	4	47	12	39	3.3%	0.28 [0.10, 0.79]
Haghighi 2017	2	18	6	20	2.2%	0.37 [0.09, 1.61]
Lei 2017	11	39	23	41	5.2%	0.50 [0.28, 0.89]
Luo 2024	10	56	11	56	4.3%	0.91 [0.42, 1.97]
Majumdar 2025	10	50	11	50	4.4%	0.91 [0.42, 1.95]
Mohib 2015	9	50	21	50	4.7%	0.43 [0.22, 0.84]
Nikolaou 2021	31	42	49	59	6.5%	0.89 [0.72, 1.10]
Owen 2024	17	64	19	64	5.3%	0.89 [0.51, 1.56]
Qiu 2023	0	25	4	25	0.8%	0.11 [0.01, 1.96]
Shah 2023	13	100	41	100	5.2%	0.32 [0.18, 0.55]
Tengberg 2016	27	33	33	39	6.5%	0.97 [0.78, 1.19]
Tian 2018	24	50	34	50	6.1%	0.71 [0.50, 1.00]
Wang 2021	2	35	7	32	2.2%	0.26 [0.06, 1.17]
Wang 2023	9	33	14	33	4.7%	0.64 [0.32, 1.27]
Zhang 2020	0	15	5	15	0.8%	0.09 [0.01, 1.51]
Zhang 2022	18	61	37	61	5.8%	0.49 [0.31, 0.75]
Zhou 2019	5	50	27	50	4.0%	0.19 [0.08, 0.44]
Zhu 2024	3	22	9	32	2.9%	0.48 [0.15, 1.59]
**Subtotal (95% CI)**		**908**		**881**	**87.2%**	**0.53 [0.39, 0.70]**
Total events	237		424			
Heterogeneity: Tau² = 0.28; Chi² = 135.93, df = 19 (P < 0.00001); I² = 86%						
Test for overall effect: Z = 4.38 (P < 0.0001)						
**4.1.2 Preoperative**						
Ma 2021	7	63	14	62	4.1%	0.49 [0.21, 1.14]
**Subtotal (95% CI)**		**63**		**62**	**4.1%**	**0.49 [0.21, 1.14]**
Total events	7		14			
Heterogeneity: Not applicable						
Test for overall effect: Z = 1.66 (P = 0.10)						
**4.1.3 Postoperative**						
Luo 2019	7	44	17	46	4.3%	0.43 [0.20, 0.94]
Yakel 2023	7	40	18	49	4.4%	0.48 [0.22, 1.03]
**Subtotal (95% CI)**		**84**		**95**	**8.7%**	**0.45 [0.26, 0.78]**
Total events	14		35			
Heterogeneity: Tau² = 0.00; Chi² = 0.03, df = 1 (P = 0.86); I² = 0%						
Test for overall effect: Z = 2.84 (P = 0.004)						
**Total (95% CI)**		**1055**		**1038**	**100.0%**	**0.52 [0.40, 0.68]**
Total events	258		473			
Heterogeneity: Tau² = 0.27; Chi² = 142.40, df = 22 (P < 0.00001); I² = 85%						
Test for overall effect: Z = 4.83 (P < 0.00001)						
Test for subgroup differences: Chi² = 0.23, df = 2 (P = 0.89), I² = 0%						

(B)

Study or Subgroup	TXA Mean	TXA SD	TXA Total	Control Mean	Control SD	Control Total	Weight	Std. Mean Difference IV, Random, 95% CI
Chen 2019	1.5	0.74	88	2.5	1.48	88	17.2%	-0.85 [-1.16, -0.54]
Nikolaou 2021	1.33	1	42	1.93	1.25	59	16.5%	-0.52 [-0.92, -0.11]
Shah 2023	1.3	0.74	100	3.2	1.41	100	17.1%	-1.68 [-2.00, -1.36]
Tengberg 2016	2.4	1.9	33	3.1	2.3	39	15.8%	-0.33 [-0.79, 0.14]
Zhang 2022	0.33	0.74	61	1	1.48	61	16.8%	-0.57 [-0.93, -0.21]
Zhou 2019	2.3	0.97	50	2.27	0.76	50	16.5%	0.03 [-0.36, 0.43]
**Total (95% CI)**			**374**			**397**	**100.0%**	**-0.66 [-1.15, -0.17]**
Heterogeneity: Tau² = 0.34; Chi² = 53.07, df = 5 (P < 0.00001); I² = 91%								
Test for overall effect: Z = 2.64 (P = 0.008)								

Supplementary Figure S5. Forest plot for the effect of tranexamic acid (TXA) versus control on (A) transfusion rate and (B) number of transfused units per patient, considering trial's arm with multiple doses of TXA in multi-arm trials.

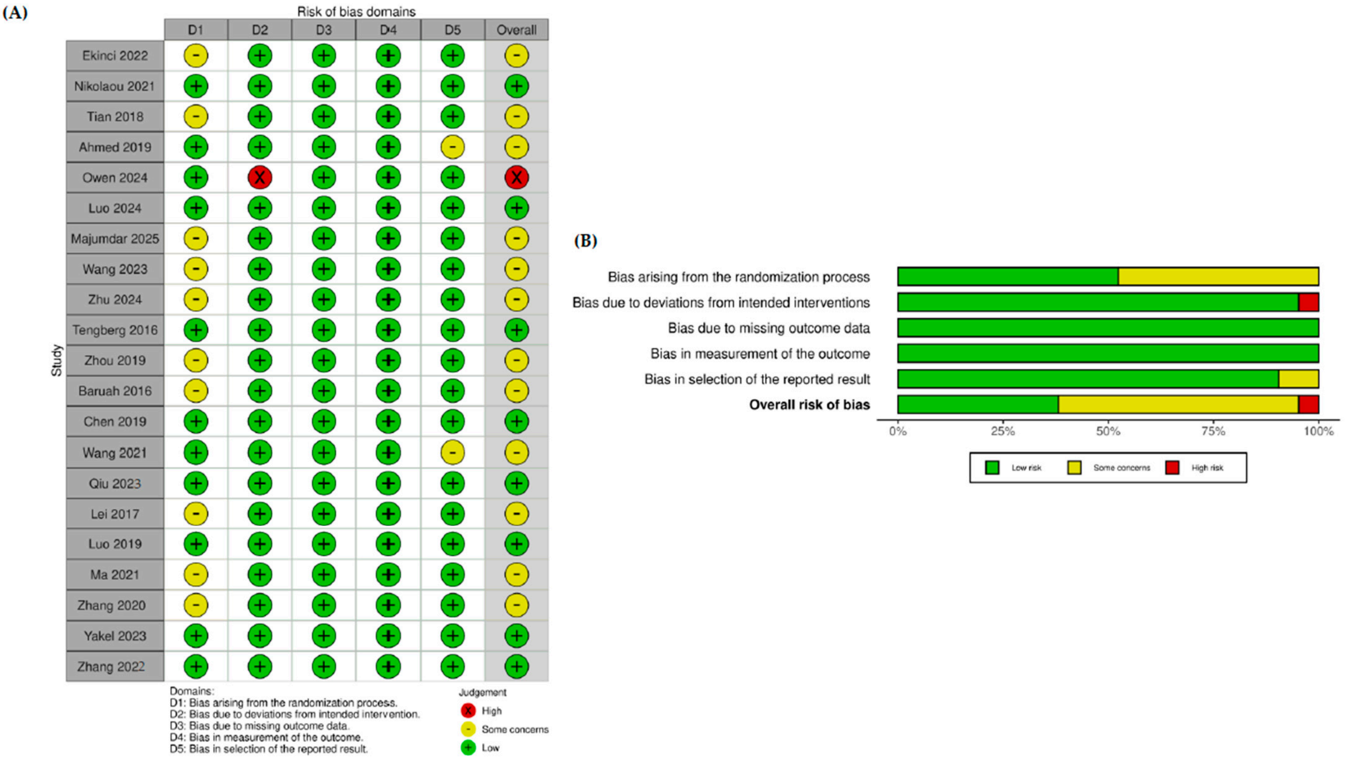

Supplementary Figure S6. Risk of bias assessment for total blood loss and hidden blood loss (A) traffic light plot and (B) summary plot.

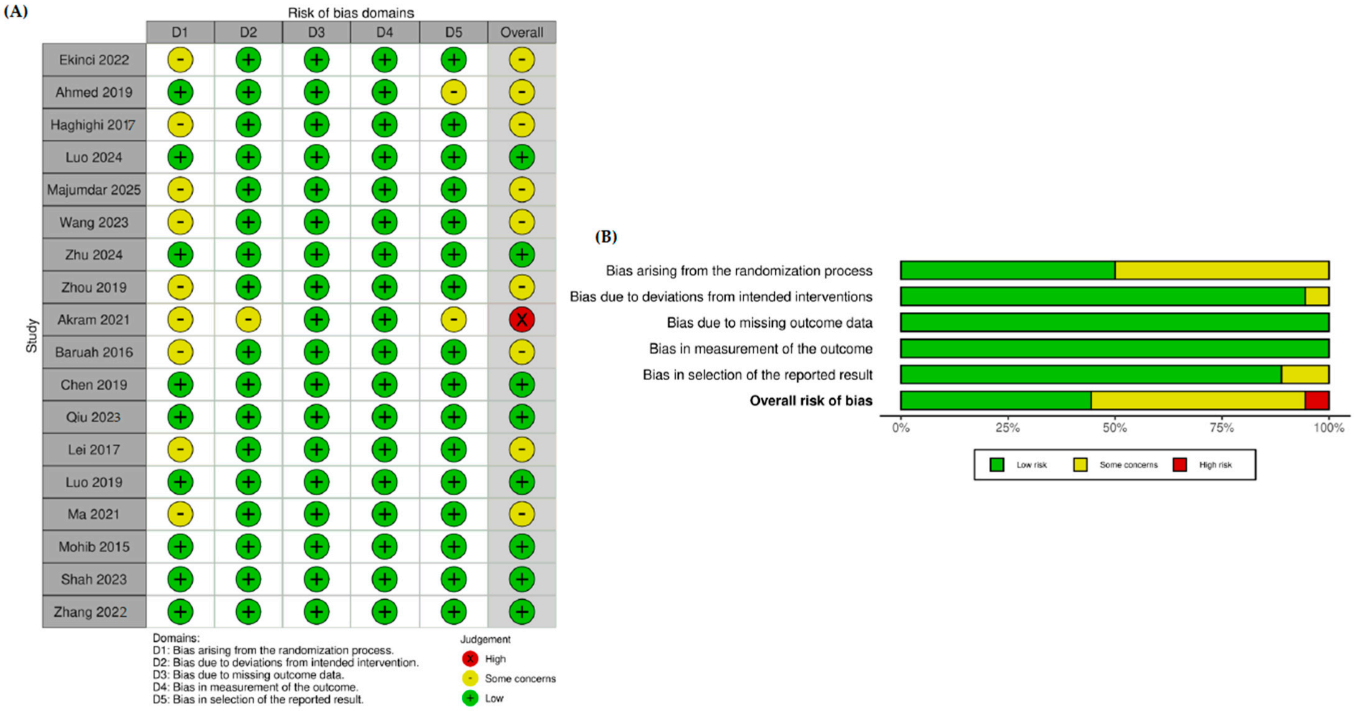

Supplementary Figure S7. Risk of bias assessment for change in hemoglobin and hematocrit (A) traffic light plot and (B) summary plot.

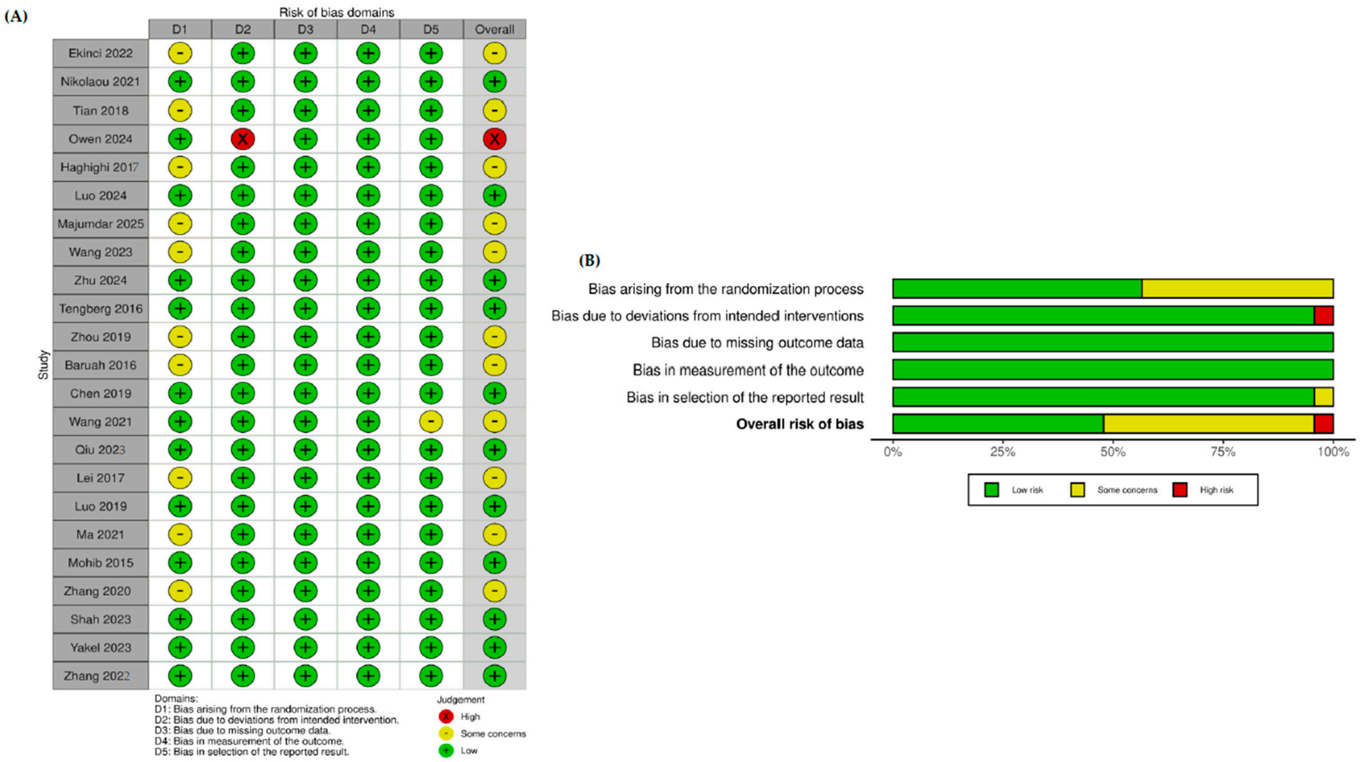

Supplementary Figure S8. Risk of bias assessment for transfusion rate (A) traffic light plot and (B) summary plot.

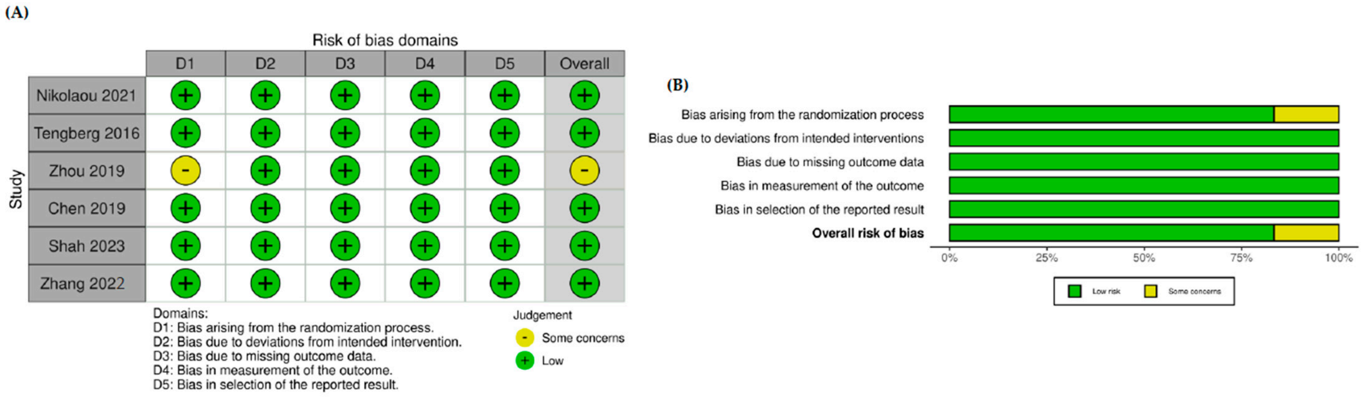

Supplementary Figure S9. Risk of bias assessment for number of transfused units per patient (A) traffic light plot and (B) summary plot.

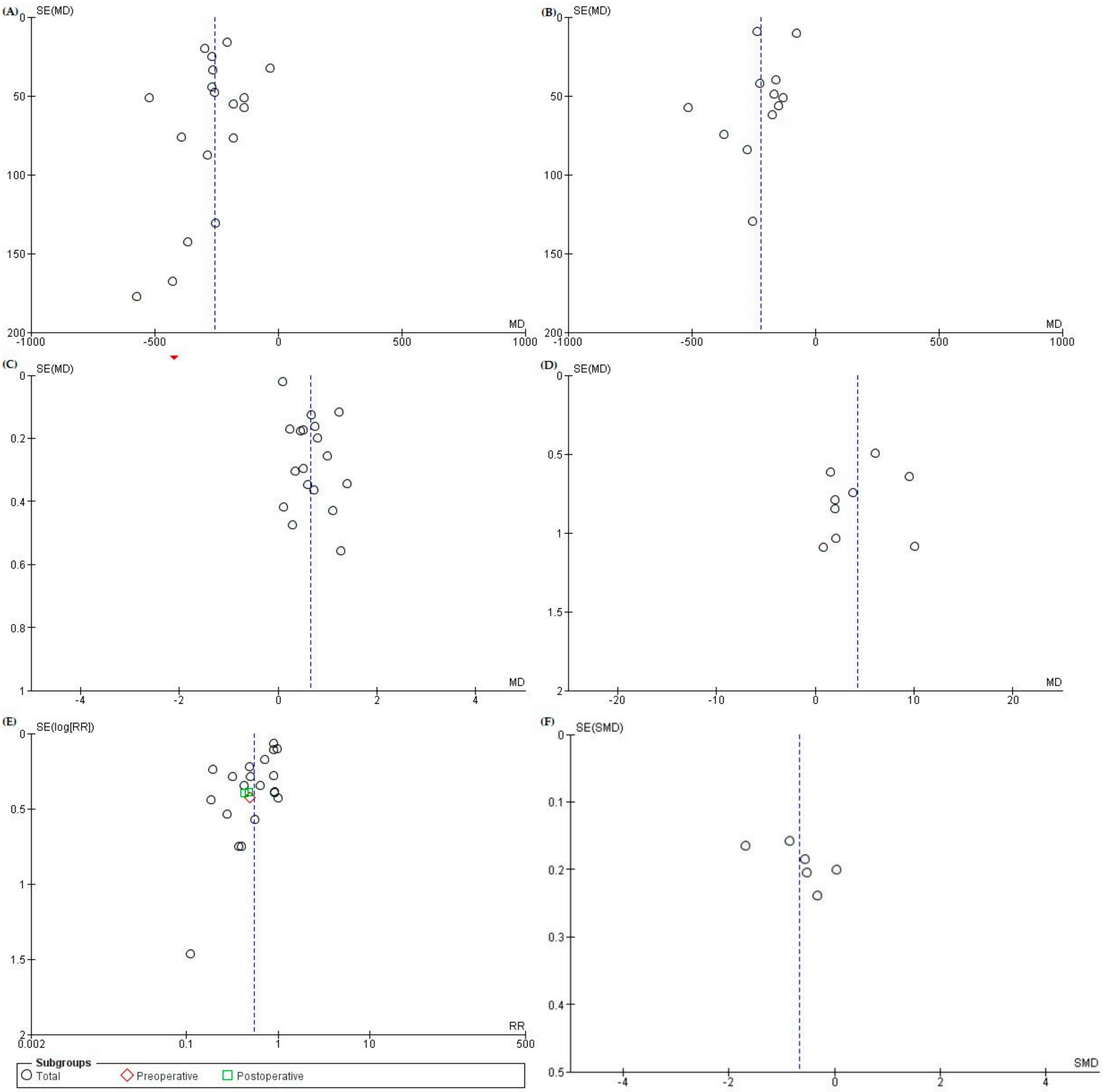

Supplementary Figure S10. Funnel plots for estimates in meta-analysis of tranexamic acid use and (A) total blood loss, (B) hidden blood loss, (C) change in hemoglobin, (D) change in hematocrit, (E) risk for transfusion and (F) number of transfused units per patient.
